# HMGR and CHS gene cloning, characterizations and tissue-specific expressions in *Polygala tenuifolia* Willd

**DOI:** 10.1371/journal.pone.0300895

**Published:** 2024-03-25

**Authors:** Yang Liu, Xiaofang Ma, Fuying Mao, Jinmiao Qiu, Jingyi Bi, Xiaowei Li, Xian Gu, Yuguang Zheng, Yunsheng Zhao

**Affiliations:** 1 College of Pharmacy, Hebei University of Chinese Medicine, Shijiazhuang, Hebei Province, China; 2 Traditional Chinese Medicine Processing Technology Innovation Center of Hebei Province, Shijiazhuang, Hebei Province, China; 3 International Joint Research Center on Resource Utilization and Quality Evaluation of Traditional Chinese Medicine of Hebei Province, Shijiazhuang, Hebei Province, China; 4 Yinchuan Women and Children Health Care Hospital, Yinchuan, Ningxia, China; 5 Experimental Center, Hebei University of Chinese Medicine, Shijiazhuang, Hebei Province, China; 6 Hebei Chemical and Pharmaceutical College, Shijiazhuang, Hebei Province, China; ICAR - Central Tobacco Research Institute, INDIA

## Abstract

Triterpenoid saponins and flavonoids have several pharmacological activities against *P*. *tenuifolia*. The 3-hydroxy-3-methylglutaryl-CoA reductase (HMGR) and chalcone synthase (CHS) are the rate-limiting enzymes of triterpenoid saponin and flavonoid biosynthesis, respectively. In this study, HMGR and CHS genes were cloned from *P*. *tenuifolia*, and their bioinformatics analyses and tissue-specific expression were investigated. The results showed that the HMGR and CHS genes were successfully cloned, separately named the PtHMGR gene (NCBI accession: MK424118) and PtCHS gene (NCBI accession: MK424117). The PtHMGR gene is 2323 bp long, has an open reading frame (ORF) of 1782 bp, and encods 593 amino acids. The PtCHS gene is 1633 bp long with an ORF of 1170 bp, encoding 389 amino acids. PtHMGR and PtCHS were both hydrophobic, not signal peptides or secreted proteins, containing 10 conserved motifs. PtHMGR and PtCHS separately showed high homology with HMGR and CHS proteins from other species, and their secondary structures mainly included α-helix and random curl. The tertiary structure of PtHMGR was highly similarity to that the template 7ULI in RCSB PDB with 92.0% coverage rate. The HMG-CoA-binding domain of PtHMGR is located at 173–572 amino acid residues, including five bound sites. The tertiary structure of PtCHS showed high consistency with the template 1I86 in RCSB PDB with 100% coverage rate, contained malonyl CoA and 4-coumaroyl-CoA linkers. The expression of PtHMGR and PtCHS is tissue-specific. PtHMGR transcripts were mainly accumulated in roots, followed by leaves, and least in stems, and were significantly positively correlated with the contents of total saponin and tenuifolin. PtCHS was highly expressed in the stems, followed by the leaves, with low expression in the roots. PtCHS transcripts showed a significant positive correlation with total flavonoids content, however, they were significantly negatively correlated with the content of polygalaxanthone III (a type of flavonoids). This study provided insight for further revealing the roles of PtHMGR and PtCHS.

## Introduction

*Polygala tenuifolia* Willd. was first recorded in the Shennong’s Herbal Classic of Materia Medica, and its dry roots are also known as Yuan Zhi [1], which have the functions of intelligence, sedation, hypnosis, expectorant, detoxification, detumescence, anti-inflammatory, neuroprotection, and anti-depression [1–4]. In China, *P*. *tenuifolia* is mainly cultivated in the north, and the Shanxi and Shaanxi provinces are traditionally regarded as the areas with good quality and high output [5,6].

Triterpenoid saponins, xanthones, oligosaccharide esters, alkaloids, and other components [6,7] have been found in *P*. *tenuifolia*. Polygala saponins are the main active components of *P*. *tenuifolia*, and its structure is mainly oleanane pentacyclic triterpenes [8], including sibiricasaponin A, sibiricasaponin B, sibiricasaponin C, sibiricasaponin E, tenuifolisaponin A, tenuifolisaponin B, polygalasaponin ⅩⅩⅦ, polygalasaponin ⅩⅩⅫ [9–11], etc. The saponins of *P*. *tenuifolia* have many pharmacological activities such as expectorant, sedative, anti-dementia activities, etc, have a significant protective effect on the mitochondrial ultrastructure, and can effectively relieve oxidative stress damage and inhibit neuronal damage by alleviating the neurotoxicity of amyloid beta-protein 1–40 (Aβ_1–40_) [12]. Polygala saponins can prevent pheochromocytoma (PC12) cell apoptosis induced by Aβ_1–40_. The endogenous apoptosis pathway is blocked by inhibiting the expression of Bcl-2-related X protein (Bax) and cytochrome c (Cyt c) and increasing the expression of b-lymphocytoma-2 (Bcl-2) and the Bcl-2/Bax ratio [13].

**Fig 1:**
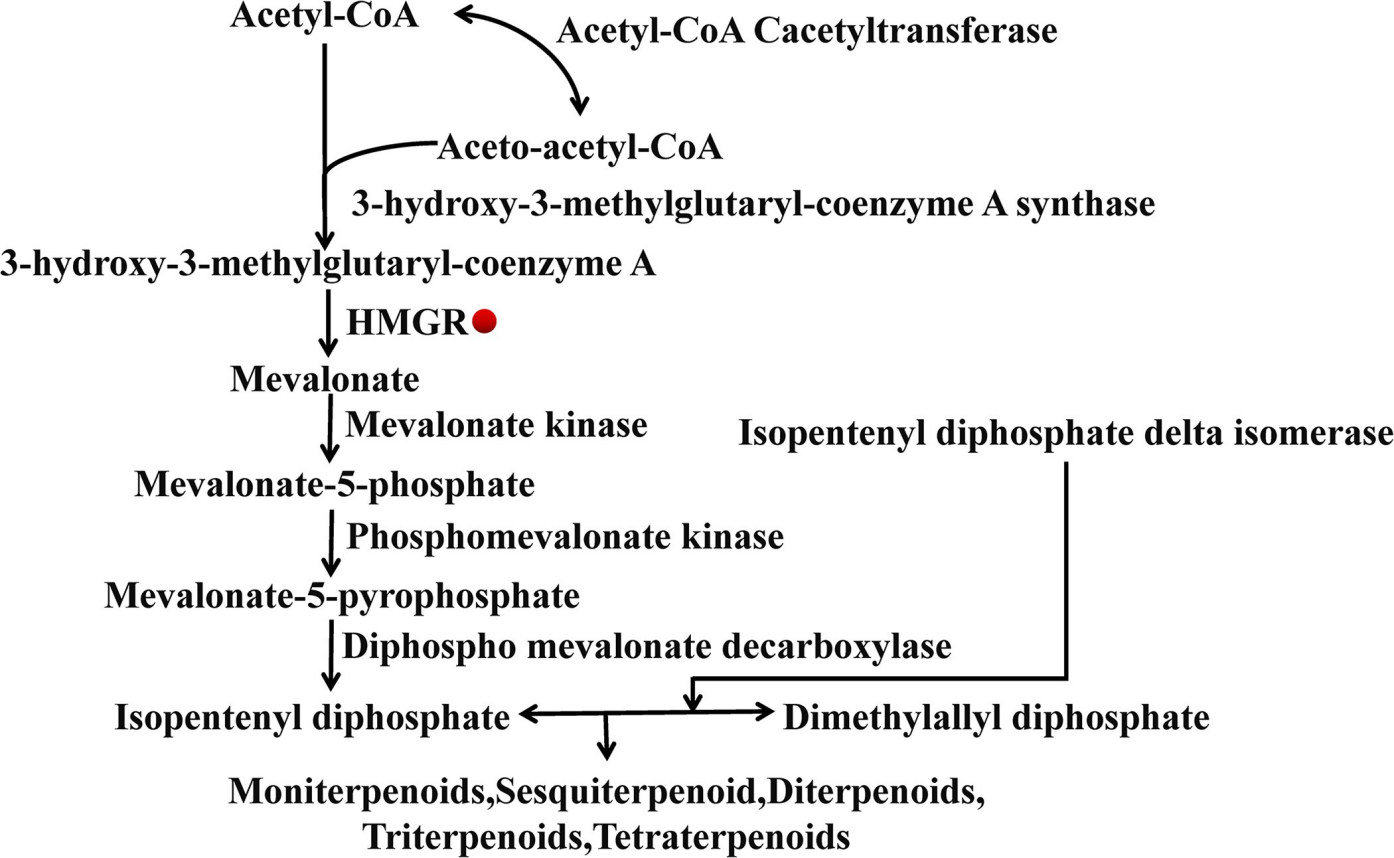
HMGR is involved in terpenoid synthesis pathway.

The cytoplasmic mevalonate pathway (MVA) uses the isopentenyl diphosphate (IPP) and dimethylallyl diphosphate (DMAPP) to synthesize various terpenoids [14] and is a well-known pathway for triterpenoid saponin synthesis [15]. 3-Hydroxy-3-methylglutaryl coenzyme A reductase (HMGR) is the first key rate-limiting enzyme in the MVA pathway [16] (Fig 1) and catalyzes the irreversible conversion of 3-hydroxy-3-methylglutaryl coenzyme A (HMG-CoA) to mevalonic acid [17]. Its expression level affects the accumulation of saponins in *P*. *tenuifolia*. To date, the HMGR genes in many plants have been successfully isolated and cloned, such as *Alisma orientale* [18], *Bunium persicum* [19], *Cucumis melo* [20], *Lithospermum erythrorhizon* [21], *Catharanthus roseus* [22], *Salvia miltiorrhiza* [23] and etc. However, the involvement of HMGR in the terpenoid biosynthetic pathway has not yet been identified in *P*. *tenuifolia*.

**Fig 2:**
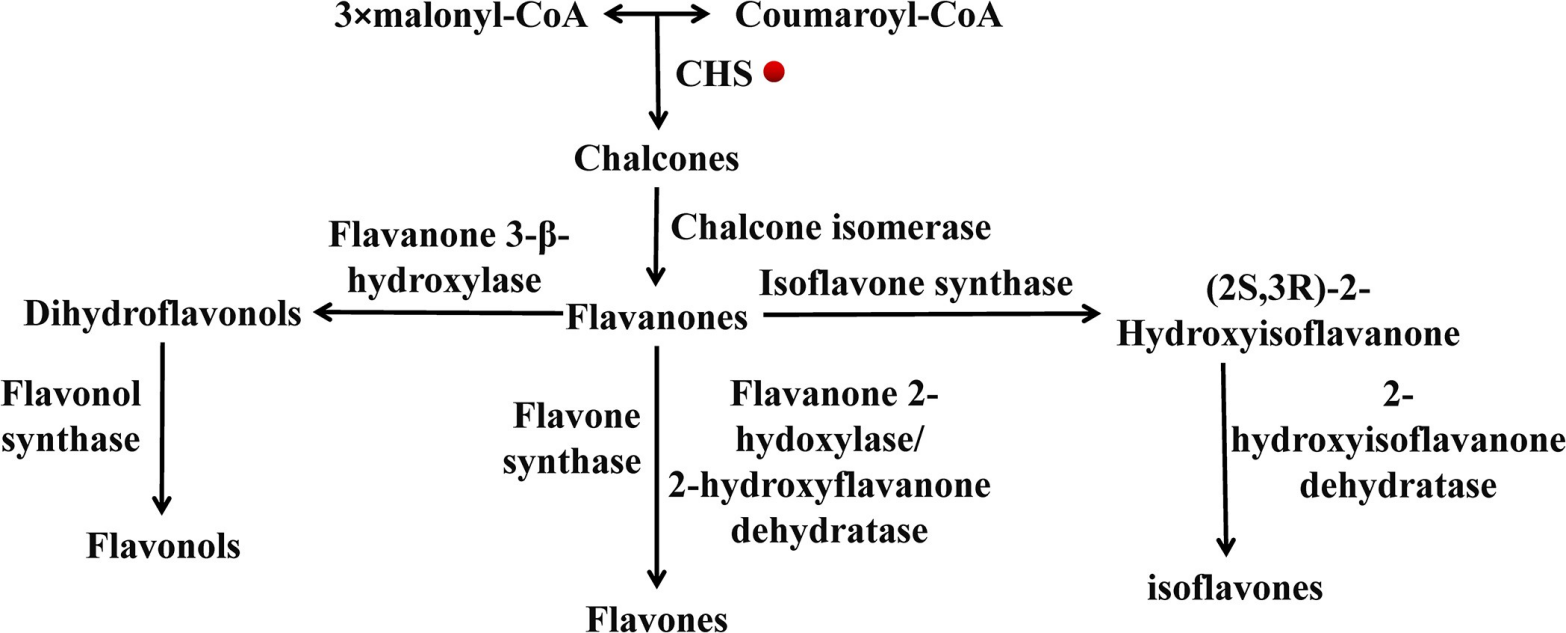
CHS is involved in flavonoids synthesis pathway.

Xanthones, also known as benzchromones or diphenyl pyrones, are a type of flavonoids with the structure of a tricyclic aromatic hydrocarbon system [24,25]. They are another important type of active components in *P*. *tenuifolia*, such as onjixanthone Ⅰ, onjixanthone Ⅱ, polygalaxanthone Ⅲ, polygalaxanthone V, polygalaxanthone Ⅵ, sibiricaxanthone A and sibiricaxanthone B [26–28], etc., and are involved in many pharmacological activities, including anti-inflammatory, bactericidal activities, etc. Chalcone synthase (CHS) plays a vital role in the flavonoid biosynthesis pathway and is a key enzyme that directs the metabolic flux of the phenylpropanoid pathway toward flavonoid biosynthesis [29] (Fig 2). It catalyzes one p-coumaroyl-CoA and three malonyl-CoA thioesters to form a chalcone. Then, flavonoids, isoflavones and anthocyanins were produced by Chalcone isomerase (CHI), Isoflavone synthase (IFS) and 2-oxo-glutarate-dependent dioxygenase (2-ODD) [30]. The formation of flavonoids depends significantly on CHS, which is regarded as the first rate-limiting enzyme involved in flavonoid synthesis [31]. It has been cloned and studied in many higher plant species, including *Oryza sativa* [32], *Carthamus tinctorius* [33], *Bletilla striata* [34], *Arabidopsis thaliana* [35] and *Actinidia eriantha* [36], etc. However, research on the CHS gene of *P*. *tenuifolia* remains unexplored.

To explore the acting mechanism of action of HMGR and CHS, the current study cloned PtHMGR and PtCHS genes from *P*. *tenuifolia*, investigated their gene sequence, conserved motifs, structure and function, and analyzed their phylogenesis, tissue-specific expression and the correlation between PtHMGR or PtCHS gene expressions and saponin or flavonoid contents, which was beneficial for revealing the functions of *PtHMGR* and *PtCHS* in the biosynthesis of saponins and flavonoids, and provided excellent genes for quality breeding in *P*. *tenuifolia*.

## Materials and methods

### Samples collection

*P*. *tenuifolia* was collected by Ma Xiaofang of Yinchuan women and Children Health Care Hospital from Rolling Bell Mouth, Helan Mountain, Yinchuan City, Ningxia Province (105°54′42″ N, 38°35′31″ E). Samples were collected using a random sampling method by randomly collecting whole plants of *P*. *tenuifolia*. Prof. Zhao Yunsheng of Hebei University of Traditional Chinese Medicine identified the samples as *Polygala tenuifolia* Willd. of the Polygalaceae family. The samples were stored at the Traditional Chinese Medicine Processing Technology Innovation Center of Hebei Province (voucher: NXPT20170723). After collecting the samples, dirt was removed from the plant surface. Take 10 g of the sample and rinse it with distilled water two or three times. Filter paper was used to absorb any surface water. The roots, stems, and leaves were placed in 5 mL sterile, enzyme-free freezing tubes, respectively. The tubes were placed in liquid nitrogen for immediate freezing. The tubes were returned to the laboratory and stored in a refrigerator at -80°C for later use. Next, a 50 g sample was taken to remove any dirt from the plant surface, and the roots, stems, and leaves were collected. The samples were dried in the sun and stored at room temperature to determine their composition. Every experimental sample, aside from the voucher specimens, was stored for one year.

### Total RNA extraction and synthesis of cDNA

The root, stem, and leaf samples of *P*. *tenuifolia* (0.1 g) were rapidly ground into powder in liquid nitrogen. The RNAprep Pure Polysaccharide Polyphenol Plant Total RNA Extraction Kit (DP441, Tiangen, China) was used to extract the total RNA. The RNA was electrophoresed through a 1.0% agarose gel in TBE buffer at 150V for 20 min. RNA samples of 2 μl were taken, and the OD_260_ and OD_280_ values were determined by the NanoDrop2000 ultramicroscopic spectrophotometer (Thermo Fisher, China), and the ratio of OD_260_/OD_280_ was calculated. Total RNA was used as a template for the synthesis of the first strand of cDNA using the RevertAid First Strand cDNA Synthesis Kit (Fermentas, China)

### Cloning the core fragments of PtHMGR and PtCHS genes

The conserved fragments of HMGR and CHS genes were obtained after these sequences of other species were aligned with Cluster-X software. Four degenerate primers, named HMGR-F1, HMGR-R1, CHS-F1, and CHS-R1 (Table 1), were separately designed using Primer Premier 5.0 software and were produced by Sangon Biotech (Shanghai) Co., Ltd. Then, the PCR amplification were performed with the following system: 5.0 μL of the first strand of cDNA, 15.0 μLof PCR-Grade Water, 25.0 μL of 2× Ex Taq Buffer (TaKaRa, China), 1.0 μL of dNTP Mix (10 mM), 1.0 μL of Ex Taq (TaKaRa, China), 1.5 μL of HMGR-F1/CHS-F1(10×), 1.5 μL of HMGR-R1/CHS-R1(10 ×). The PCR cycling process was 94°C 2 min, 1 cycle; 94°C 30 s, 55°C 30 s, 72°C 1 min, 35 cycles; 72°C 10 min, 1 cycle. The E.Z.N.A.® Gel Extraction Kit (OMEGA, USA) was used to purify the target fragment, which was then sequenced at Sangon Biotech Co., Ltd. (Shanghai, China)

**Table 1:** The primers to amplify the full-length PtHMGR and PtCHS genes.

Sl. no	Gene	Purpose of primers	Primers	Sequence (5′ to 3′)	Length
1	PtHMGR	Gene Clone	HMGR-F1	ACMGNTGGCGBGAVAAGAT	19
2			HMGR-R1	CCTTTNGANACCATGTTCATCC	22
3			HMGR-F2	ATGAACGGCGAACCCCA	17
4			HMGR-R2	GGTCTAGCAAGGATATGT	18
5		5′RACE	HMGR-GSP1	AGCTCATCATCAGACG	16
6			HMGR-GSP2	CCGAGAGGTAATGGGATG	18
7			HMGR-GSP3	AGAGAAACAATAGCAGCGAT	20
8		3′RACE	HMGR-1	GTGTTGCTTAGAGATGGGATGACGAG	26
9			HMGR-2	CCTGTTGTTCGGTTTCCTACTGCCAT	26
10	PtCHS	Gene Clone	CHS-F1	CHATHGCTCCAGAYAGTGA	19
11			CHS-R1	ARNACRCAHGCACTTGACAT	20
12			CHS-F2	ATGGTGGCTGTCAATGAA	18
13			CHS-R2	CTAGGTTGAAACACTGTG	18
14		5′RACE	CHS-GSP1	TTGGGAAGCTGATACA	16
15			CHS-GSP2	CTTCTGGAATTGGGTCTG	18
16			CHS-GSP3	TCCAACTATTACAGCAGC	18
17		3′RACE	CHS-1	GATTGGAACTCACTCTTCTGGGCT	24
18			CHS-2	AGCGAAGCTAGGCCTTAACCCTGAGA	26

### Cloning 5′ and 3′ sequences of PtHMGR and PtCHS genes

HMGR and CHS gene core fragments were used to design six 5′ Rapid Amplification of cDNA Ends (5’ RACE) specific primers (HMGR-GSP1, HMGR-GSP2, HMGR-GSP3, CHS-GSP, CHS-GSP2, and CHS-GSP3) and four 3′ Rapid Amplification of cDNA Ends (3’ RACE) specific primers (HMGR-1, HMGR-2, CHS-1, and CHS-2) (Table 1). Primer Premier software (version 5.0) was used for the primer design. The 5’ and 3’ sequences of PtHMGR and PtCHS were separately cloned using the Rapid Amplification of cDNA Ends kit (Version 2.0, Invitrogen, Shanghai) and the SMARTerTM RACE cDNA Amplification Kit (Clontech, USA). The target fragment was purified with the E.Z.N.A.® Gel Extraction Kit, and linked to the pMD®18-T vector (TaKaRa, China). Subsequently, it was transformed into DH5α *Escherichia coli* competent cells (Tiangen, China). The positive DH5α with target gene was screened and subsequently sent to Sangon Biotech Co., Ltd. (Shanghai, China) for sequencing.

### Cloning Full-length sequences of PtHMGR and PtCHS genes

Based on the PtHMGR and PtCHS gene core fragments, 5’ RACE and 3’ RACE sequences, the full-length sequences of the two genes were spliced, and the open reading frame (ORF) of the genes was predicted and verified by NCBI gene alignment analysis. Four primers of HMGR-F2, HMGR-R2, CHS-F2 and CHS-R2 (Table 1), were designed, and the ORFs of HMGR and CHS were covered. The amplification system of the full-length gene was as follows: 15.0 μL of PCR-Grade Water, 25.0 μL of 2×Ex Taq Buffer (TaKaRa, Japan), 1.0 μL of dNTP Mix (10mM),1.0 μL of Ex Taq (TaKaRa, Japan), 5.0 μL of the first strand of cDNA, 1.5 μL of primer F (10×), 1.5 μL of primer R(10×). The PCR cycling process was 94°C 2 min, 1 cycle; 94°C 30 s, 55°C 30 s, 72°C 90s, 35 cycles; 72°C 10 min, 1 cycle. The target fragment was purified, coupled with the pMD®18-T vector, and transformed into DH5α competent cells. The positive DH5α with target gene was screened and subsequently sent to Sangon Biotech Co., Ltd. (Shanghai, China) for sequencing.

### Sequence analysis of PtHMGR and PtCHS

Full-length sequences of PtHMGR and PtCHS were submitted to GenBank (http://www.ncbi.nlm.nih.gov/genbank). SnapGene 6.0.2 was used for the translation and cleavage sites analysis of PtHMGR and PtCHS. ProtParam (https://web.expasy.org/ protparam/) was used to analyze the properties of the deduced amino acid sequences.

### Conserved motifs analysis of PtHMGR and PtCHS

Multi-sequence comparison of PtCHS and PtHMGR with those of other plants was performed using Clustal W default parameters of MEGA-11 software. MEME suite 5.5.1 (https://meme-suite.org/meme/index.html) was used to analyze the conserved protein motifs of PtCHS and PtHMGR, and the predicted number was set to 10.

### Phylogenetic analysis of PtHMGR and PtCHS

Multiple amino acid sequences of PtHMGR, PtCHS, and the corresponding genes in other plants were analyzed using DNAMAN V9.0. Sequence alignment of PtHMGR and PtCHS genes was performed using the Clustal W tool in MEGA-11 software. The phylogenetic tree were constructed using the maximum likelihood (ML) (bootstrap = 1000) of MEGA-11 [37], and the phylogeny was visualized using Evolview software (http://www.evolgenius.info/evolview/#/tree view).

### Protein properties and model analysis of PtHMGR and PtCHS

BLAST (https://blast.ncbi.nlm.nih.gov/Blast.cgi) was used to search for the nucleotide and protein sequences of HMGR and CHS, and to compare their homology. The protein signal peptide prediction of PtHMGR and PtCHS were performed with the SignalP 4.1 server (http://www.cbs.dtu.dk/services/SignalP/). The TMHMM server v2.0 (http://www.cbs. dtu.dk/services/TMHMM-2.0/) were used to predict the transmembrane helices of PtHMGR and PtCHS. The secondary structures of the PtHMG and PtCHS proteins were obtained using SOPMA (http://expasy.org/tools/SOPMA). The secondary structure and solvent accessibility of the PtHMGR and PtCHS proteins were analyzed by PredictProte (https://predictprotein.org/). The secondary structures of PtHMGR and PtCHS were modified using PyMOL software. SWISS-MODEL (https://swissmodel.expasy.org) was used to predict the tertiary structures of PtHMGR and PtCHS using the 7uli.1.D 3-hydroxy-3-methylglutaryl coenzyme A reductase 1 or 1i86.1.A CHALCONE SYNTHASE 2 as the model.

### Tissue-specific expression analysis of PtHMGR and PtCHS genes

The RNA of the roots, stems or leaves of *P*. *tenuifolia* was extracted and the first cDNA strand was synthesized. Quantitative PCR primers (HMGR actin F, HMGR actin R, HMGR-F, and HMGR-R; CHS actin F, CHS actin R, CHS-F, and CHS-R) were designed and synthesized (Table 2) by Sangon Biotech Co., Ltd (Shanghai, China). The Power SYBR® Green PCR Master Mix (Applied Biosystems ® Cat. No. 4367659, Thermo Fisher, China) was used for Real-time qPCR (RT-qPCR). The reaction system contains cDNA of root, stem or leaf 5.0 μL, HMGR-F/CHS-F 0.5 μL, HMGR-R/CHS-R 0.5 μL, ddH_2_O 4.0 μL, 2×SYBR® Green PCR Master Mix 10.0 μL. The cycling program of the PCR was 95°C 3 min, 1 cycle, and 94°C 10 s, 55°C 20 s, 72°C 20s, 35 cycles; with melting curve of 65°C - 95°C. Each reaction was repeated thrice. Livak KJ’s method was used to calculate the relative gene expression. PtHMGR and PtCHS gene expression levels were normalized to the actin expression levels in each sample [38].

**Table 2:** The primers used for RT-qPCR experiment in *Polygala tenuifolia* Willd.

No.	Gene	Primers	Sequence (5′ to 3′)	Length
1	PtHMGR	HMGR actin F	CAATCCCAAGGCTAATCGTGA	21
2		HMGR actin R	CCCGAATCCAGCACAATACC	20
3		HMGR-F	AACGCCAACACAGTTAAACGA	21
4		HMGR-R	TTCCCCGTCAATCTCTGTACC	21
5	PtCHS	CHS actin F	CAATCCCAAGGCTAATCGTGA	21
6		CHS actin R	CCCGAATCCAGCACAATACC	20
7		CHS-F	CCTGCAACAATCTTCGCCAT	20
8		CHS-R	TTGGAACTGTCACACATGCG	20

### Determination of total saponin and total flavonoid contents

Senegenin and Rutin were bought from Yuanye Bio-Technology Co., Ltd. (Shanghai, China).

Preparation of total saponin standard curves: Senegenin was accurately weighed and diluted to 0.035, 0.042, 0.049, 0.056, 0.063 and 0.07 mg/mL in methanol, and 80 μL of them was separately put into 6 test tubes with stoppers, heated in water bath and evaporated to dryness. Next, 0.2 mL of 5% vanilla acetic acid and 0.8 mL of perchloric acid were added to these tubes, sealed, and heated for 15 minutes in a 60°C water bath, then taken out and cooled for 5 minutes in ice water, 5 mL of acetic acid was added, kept for 15 minutes. The absorbance of the senegenin solutions at different concentrations was determined by an uv-vis spectrophotometer (Shimadzu, Japan) at 587 nm. Then, a standard curve was drawn with the contents of the reference solutions of senegenin as the x-axis and their absorbances as the y-axis.

Preparation of total saponin test solution: 1g of dried powder of roots, stems, or leaves from *P*. *tenuifolia* was accurately weighed and placed in a round-bottom flask with 100 mL petroleum ether, degreased for 4 h, then the petroleum ether was removed, 25 mL of 75% ethanol was added to the drug residue, next, the ultrasonic extraction was performed for 2 hours, the mixture was filtered by suction, the filtrate was concentrated to the extractum, whereafter it was dissolved with methanol and set volume to 25 mL in a volumetric flask.

Preparation of total flavonoid standard curves: Rutin was precisely weighed and diluted to 0.025, 0.05, 0.075, 0.1, 0.125, 0.15 mg/mL with 70% ethanol. 1 mL of each sample was added separately to a conical flask with a stopper. 0.7 mL of 5% NaNO_2_ was added to each flask, and kept for 6 minutes, 0.7 mL of 10% Al(NO_3_)_3_ was added and stayed for 6 minutes, after 5 mL of 1 mol/L NaOH was added into the flask. The mixture was diluted to 25ml with 70% ethanol and stayed for 12 minutes. The absorbance of the mixtures with different rutin concentrations was measured at 489 nm by an uv-vis spectrophotometer. Then, the standard curve was drawn with the rutin reference solution contents as the x-axis and their absorbances as the y-axis.

Preparation of total flavonoid test solution: 1g of dried powder of roots, stems, or leaves from *P*. *tenuifolia* was accurately weighed and placed in a conical flask with 25 mL of 75% ethanol, the mixture was weighed, and extracted with the ultrasonic processing for 2 hours, then weighed again after cooling. 70% ethanol was used to make up the lost weight, the extractive was filtered and the filtrate was diluted to 25mL in a volumetric flask.

### Determination of tenuifolin and polygalaxanthone III contents

Preparation of chromatographic conditions: The tests of tenuifolin and polygalaxanthone III were separately conducted on an Agilent 1260 series high performance liquid chromatography (HPLC) system (Agilent Technologies Inc., California, USA) with a Diamonsil C_18_ column (4.6 mm × 250 mm, 5 μm). Their separation was performed under the following conditions: column temperature, 25°C; injection volume, 20 μl; flow rate, 1 mL/min. The separation of tenuifolin was performed for 20 min at 210 nm detection wavelength with the mobile phase of methanol and 0.05% phosphoric acid solution (70:30). The separation of polygalaxanthone III was carried out for 30 min at 320 nm detection wavelength with the mobile phase of acetonitrile and 0.05% phosphoric acid solution (18:82). All determinations were done with isocratic elution in triplicate.

Establishment of standard curves: Tenuifolin and polygalaxanthone III were purchased from the National Institutes for Food and Drug Control (Beijing, China). Tenuifolin was accurately weighed and diluted to 0.025, 0.05, 0.1, 0.2, 0.3, 0.4 mg/mL with methanol and then precisely weighed and diluted to 0.029, 0.058, 0.116, 0.232, and 0.464 mg/mL with methanol. After the reference solutions were measured according to the above chromatographic conditions, the standard curves were established with the tenuifolin or polygalaxanthone III contents as the x-axis and their absorbances as the y-axis.

Preparation of tenuifolin test solution: 1g of dried powder (sifting through a 50 mesh sieve) of roots, stems, or leaves from *P*. *tenuifolia* was precisely weighed and put into a conical flask with 50mL of 70% methanol. The mixture was weighed and ultrasonicated for 1 hour, then weighed again after cooling, 70% methanol was used to make up the lost weight. The mixed solution was shaken well and filtered, and the continued filtrate was taken 25mL to a round-bottom flask, dried, and refluxed with 50 ml of 10% sodium hydroxide solution for 2 h. After cooling, the pH value of the solution was adjusted to 4–5 with hydrochloric acid. Thereafter, the solution was extracted thrice with 50 ml water-saturated n-butyl alcohol. The extract solution was combined and dried. The residue was diluted to 25 mL with methanol in a volumetric flask.

Preparation of polygalaxanthone III test solution: 1g of dried powder of roots, stems or leaves from *P*. *tenuifolia* was accurately weighed and placed in a conical flask with 25mL of 70% methanol, the mixture was weighed and refluxed for 2 hours, then weighed again after cooling, 70% methanol was used to make up the lost weight, the extractive was filtered and the filtrate was diluted to 25mL in a volumetric flask.

Above reference and test solutions were all filtered through 0.22 μm microfiltration membrane before HPLC determination.

### Data analysis

Data were processed by IBM SPSS Statistics 22.0 software (International Business Machines Corporation, USA), where one-way analysis of variance (ANOVA) as well as LSD pairwise comparisons were performed between different plant tissues. P values less than 0.05 and 0.01 were considered as statistical significance and great significance. All values are shown as the mean value ± standard deviation. The data were visualized by Excel 2019 (Microsoft Inc., Redmond,WDC, USA) and Graphpad Prism 8 software (Dotmatics, USA).

## Results and discussion

### Isolation of PtHMGR and PtCHS genes

**Fig 3:**
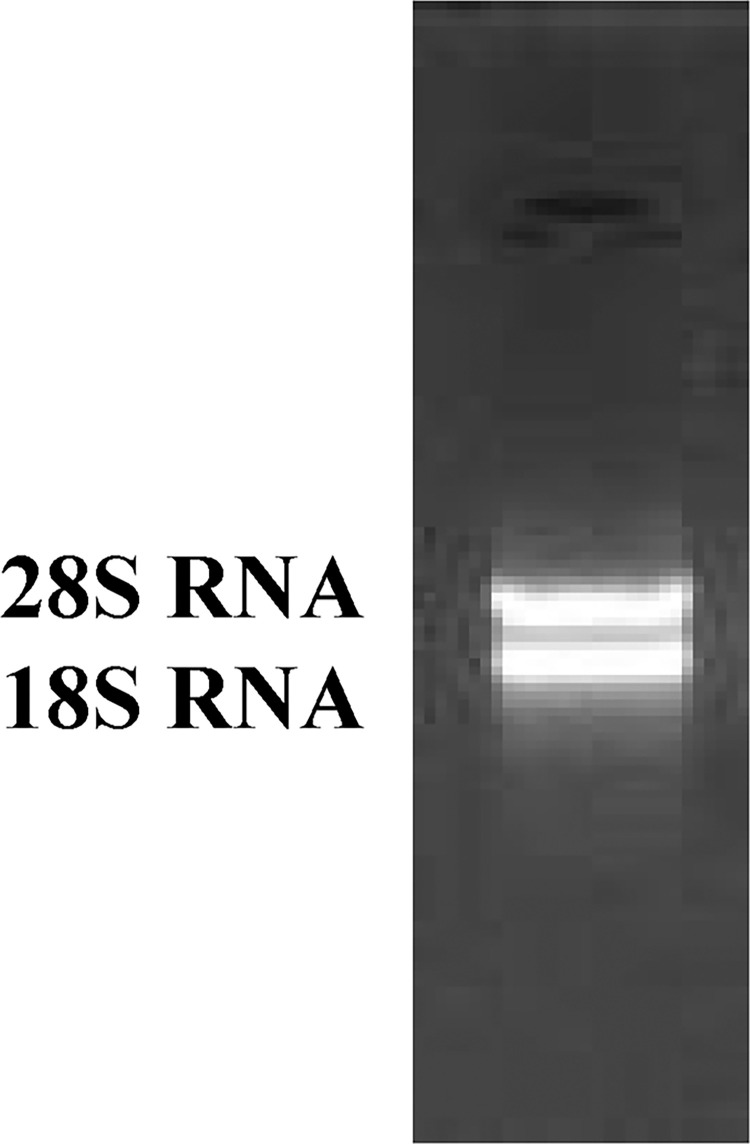
Total RNA electropherogram of *P*. *tenuifolia* root.

**Fig 4:**
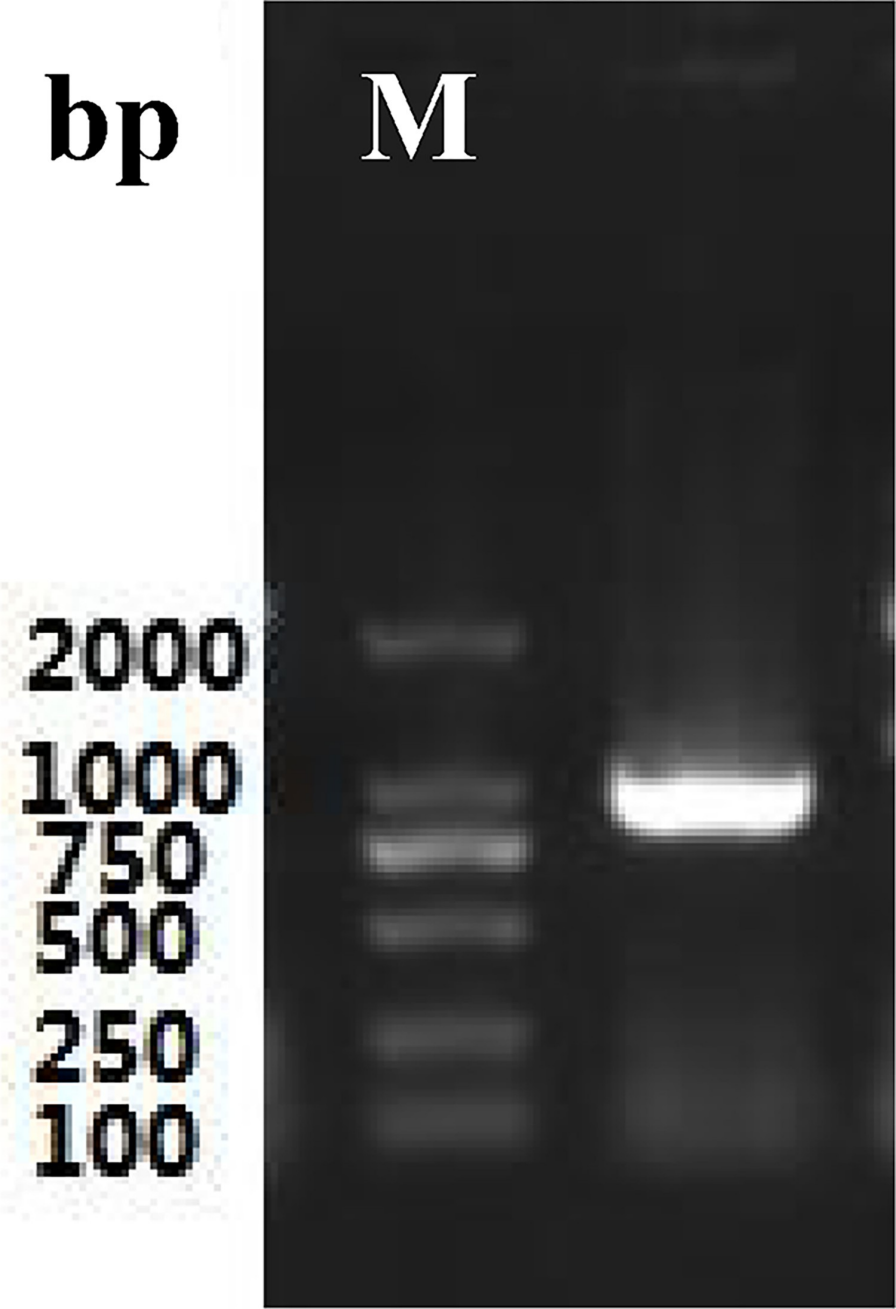
The electropherogram of intermediate fragments of PtHMGR gene.

**Fig 5:**
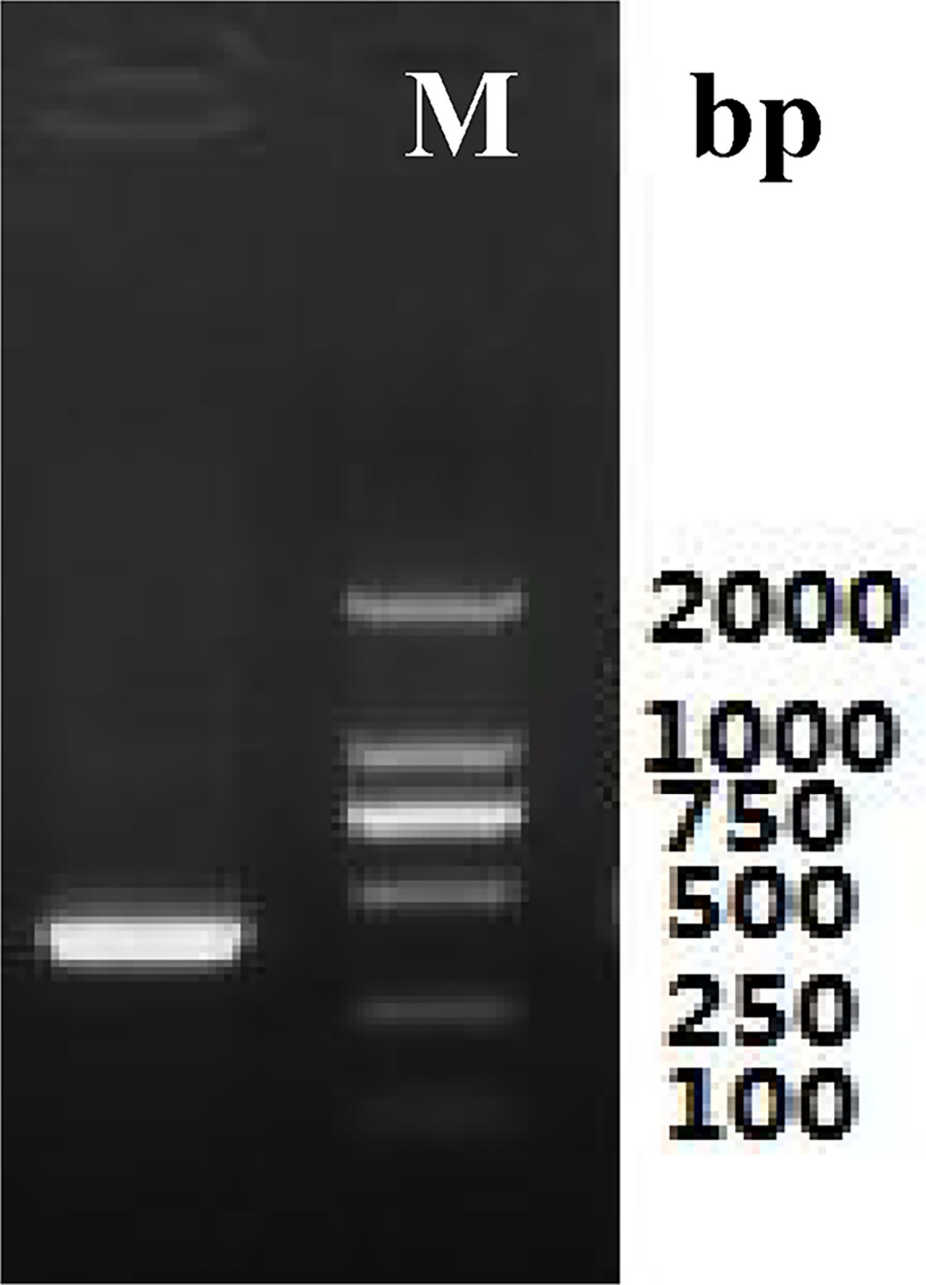
The electropherogram of intermediate fragments of PtCHS gene.

**Fig 6:**
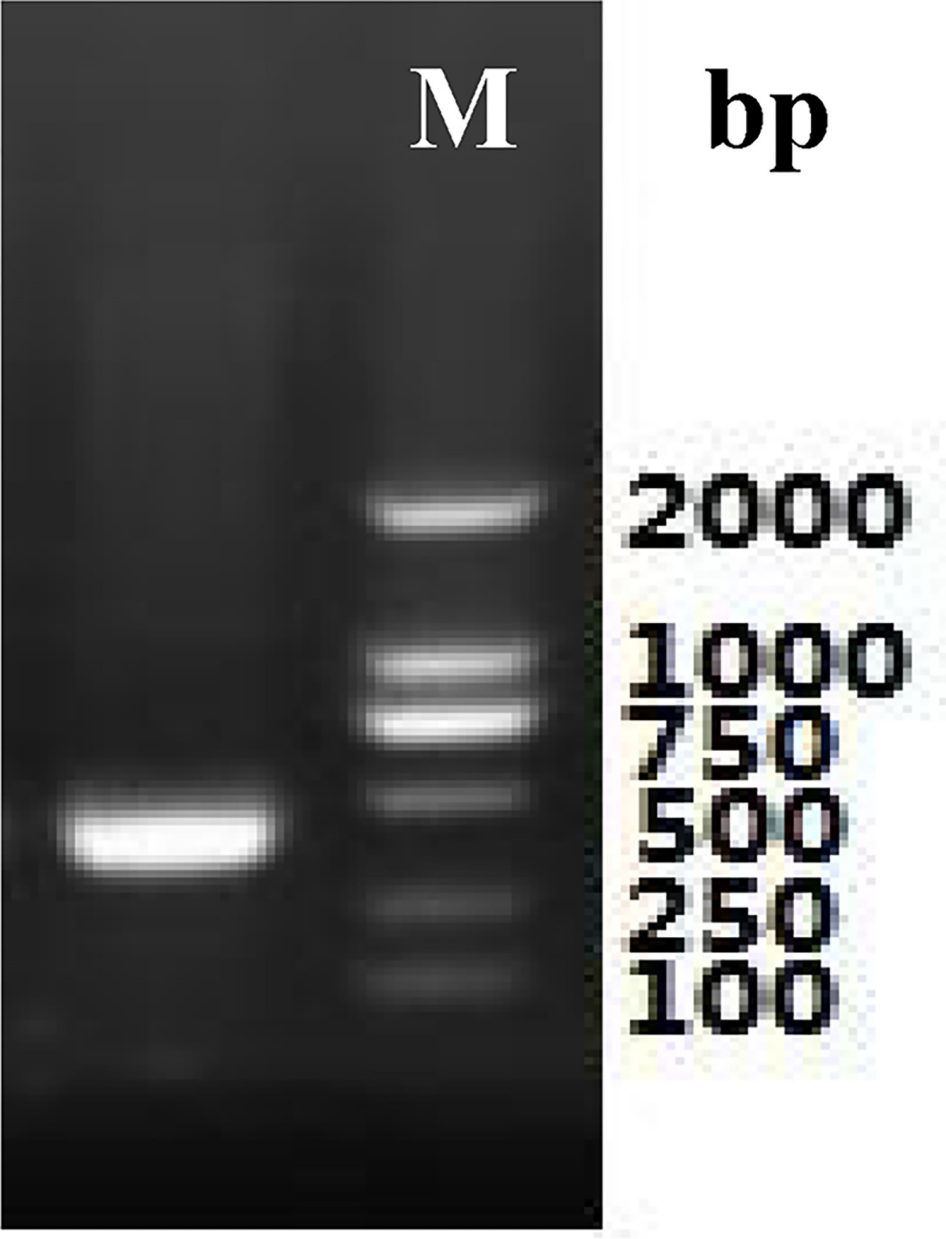
5’RACE electrophoretogram of PtHMGR gene.

**Fig 7:**
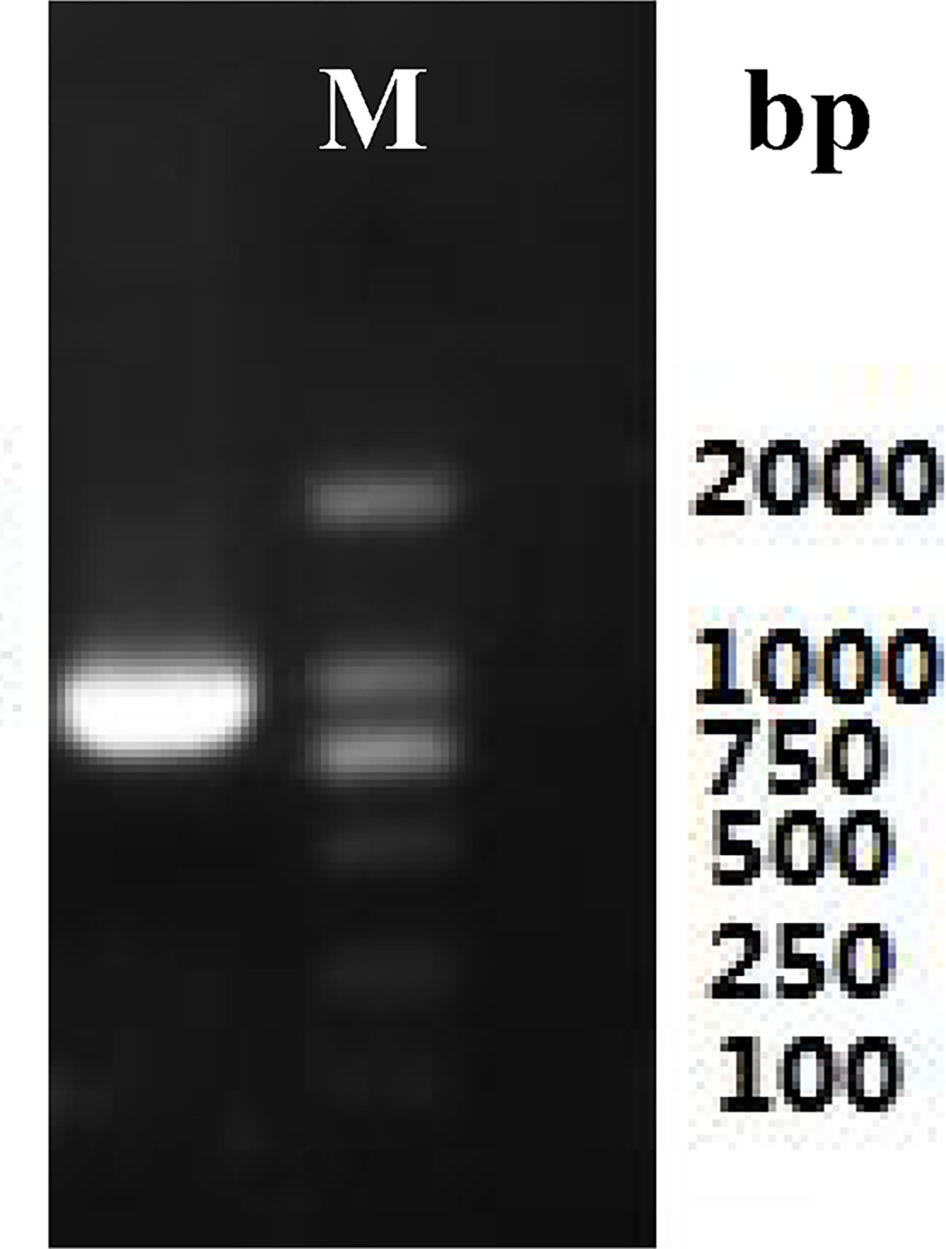
5’RACE electrophoretogram of PtCHS gene.

**Fig 8:**
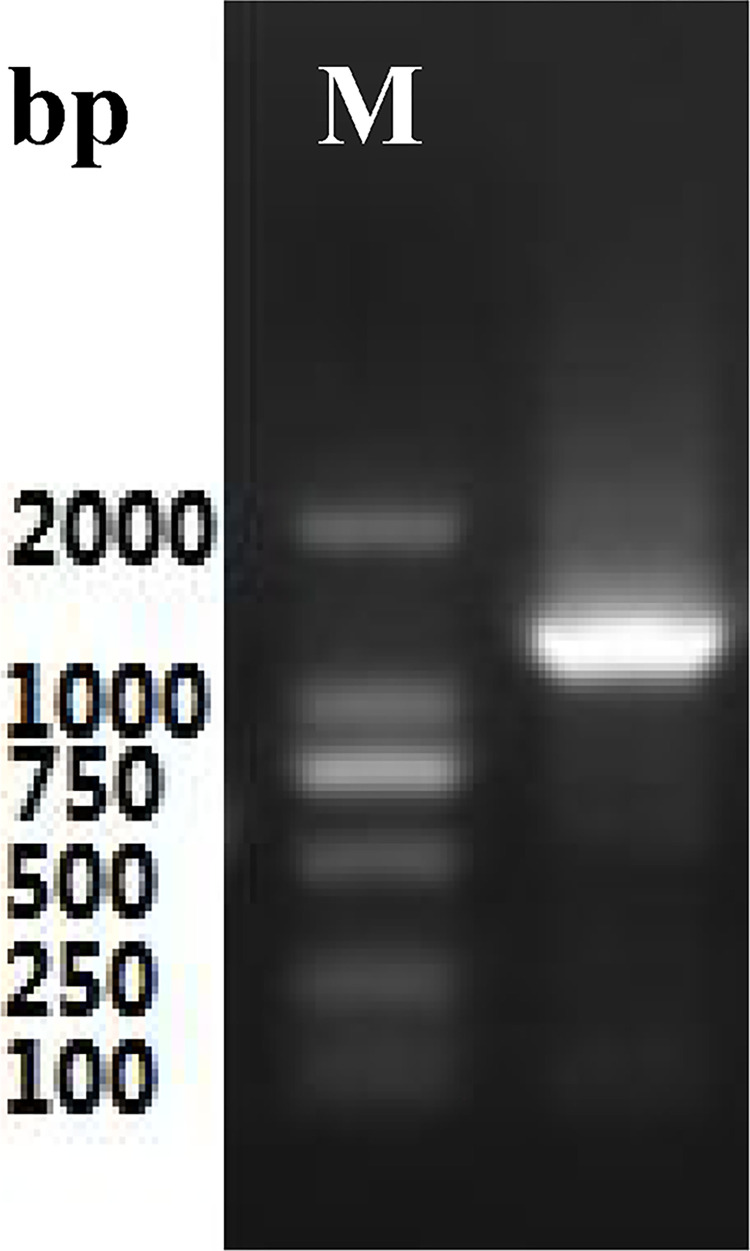
3’RACE electrophoretogram of PtHMGR gene.

**Fig 9:**
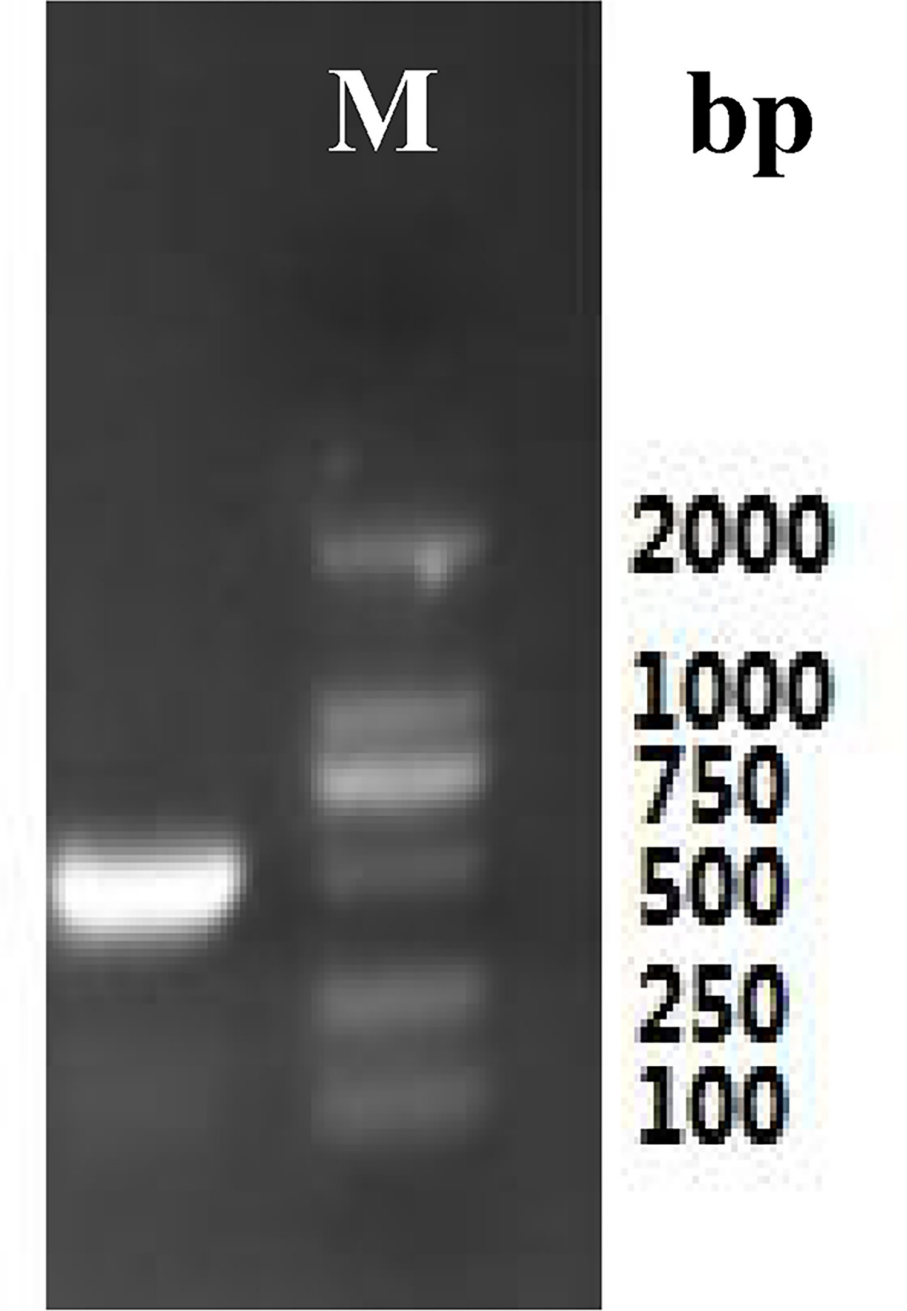
3’RACE electrophoretogram of PtCHS gene.

**Fig 10:**
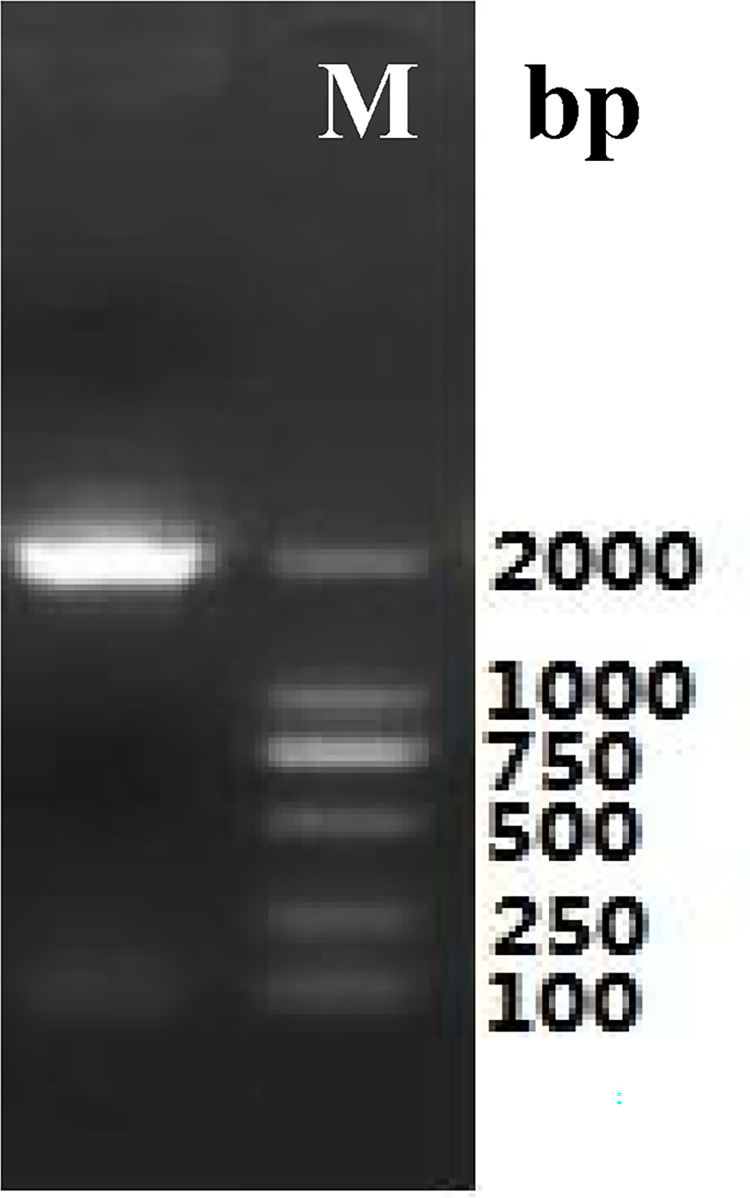
The electropherogram of full length fragments of PtHMGR gene.

**Fig 11:**
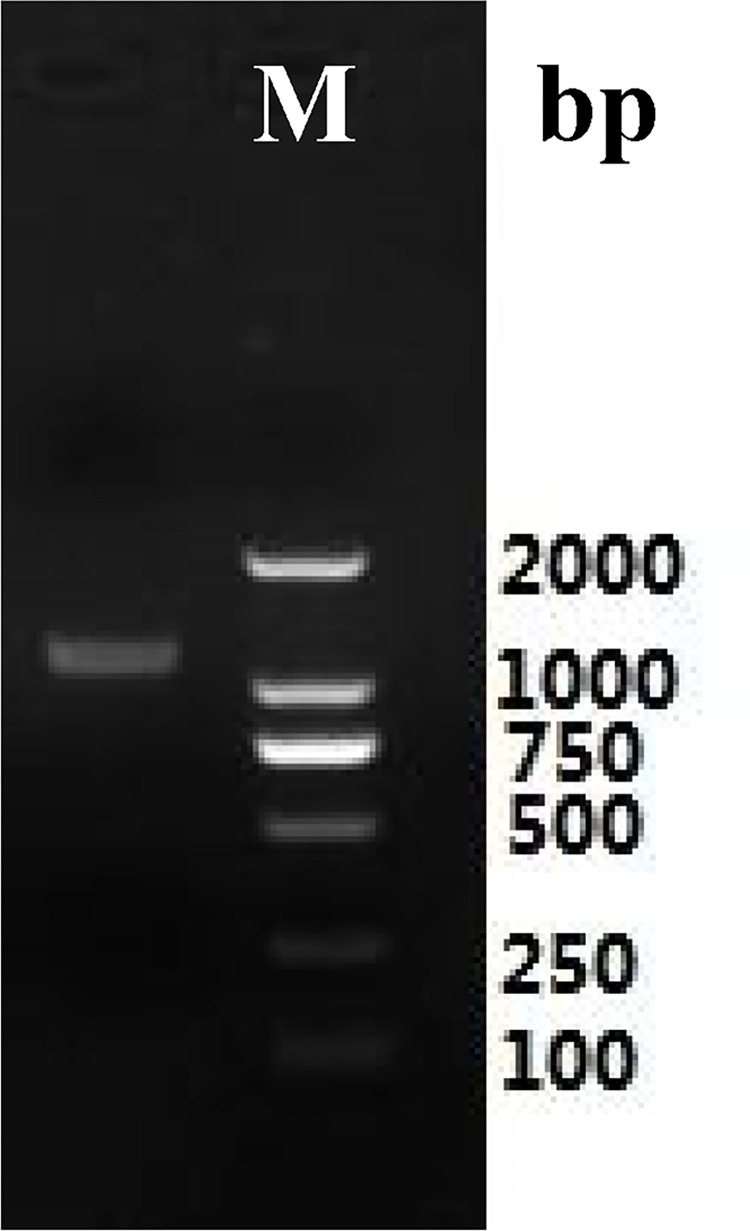
The electropherogram of full length fragments of PtCHS gene.

Total RNA (Fig 3) was extracted from the roots of *P*. *tenuifolia* and successfully reverse-transcribed into cDNA. The OD260/OD280 value of RNA was 1.94, indicating low contamination and high purity [39], and the RNA yield was more than 1 mg/mL. After cloning, the lengths of the PtHMGR and PtCHS core fragments were 925 bp and 425 bp, respectively (Figs 4 and 5). Then, 5’ and 3’ RACE (Figs 6–9) PCR generated 456 bb and 1254 bp fragments for PtHMGR, 942 bp and 464 bp fragments for PtCHS, respectively. The full-length sequences of the PtHMGR and PtCHS genes were joined according to the core fragments, 5’ and 3’ fragments, and four primers, HMGR-F2, HMGR-R2, CHS-F2, and CHS-R2 (Figs 10 and 11). The complete sequences of PtHMGR and PtCHS were cloned and recorded in the NCBI database (PtHMGR Accession No. MK424118; and PtCHS Accession No.: MK424117). HMGR and CHS separately participate in the biosynthesis of terpenoids and flavonoids in plants, and are the first rate-limiting enzymes in the MVA pathway and flavonoid synthesis pathway, respectively [32,40]. Original gel image at S1 Raw images in the Supporting Information.

### Sequence analysis of PtHMGR and PtCHS

**Fig 12:**
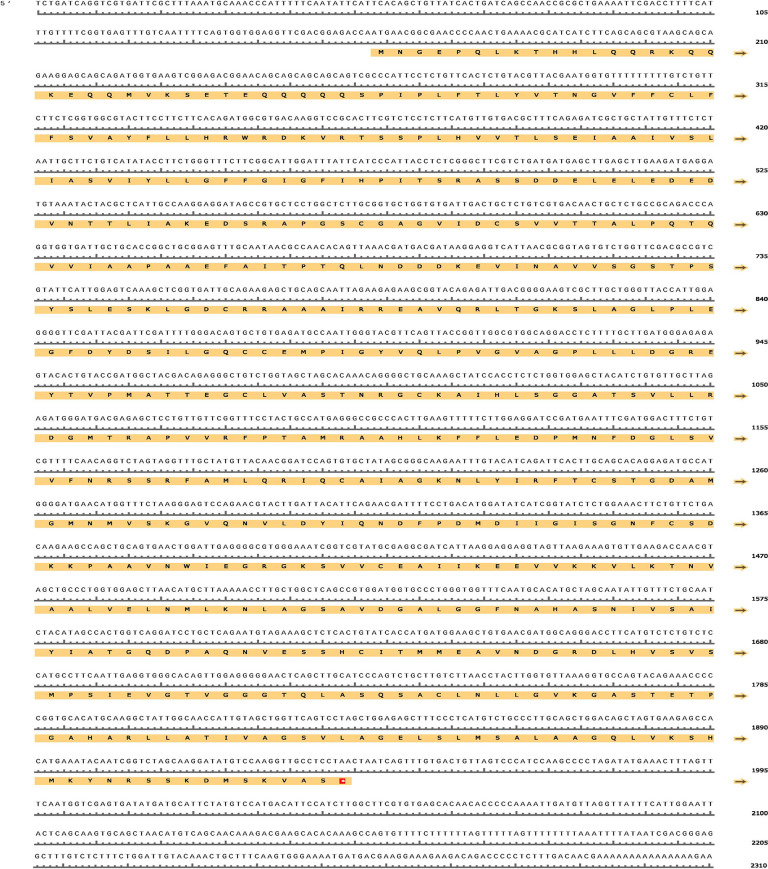
Full-length cDNA and deduced amino acid sequence of PtHMGR. A red square box highlights the initial codon and the stop codon.

**Fig 13:**
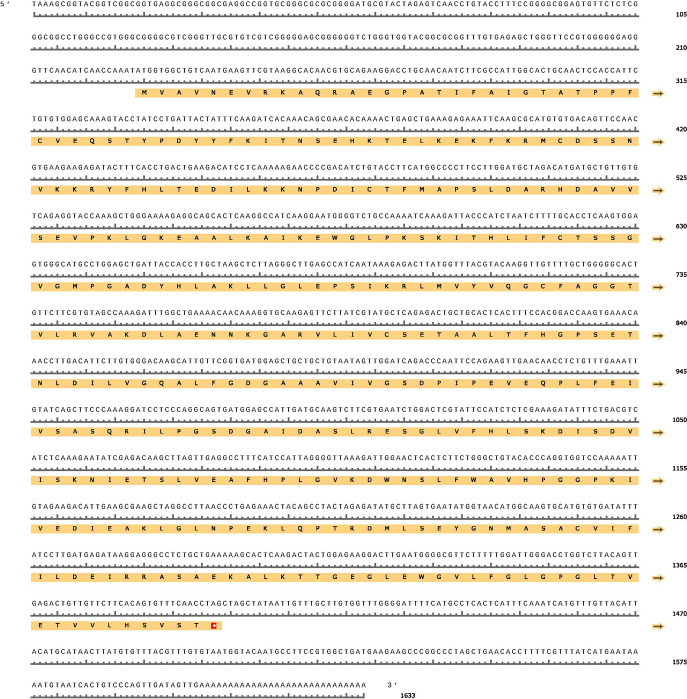
Full-length cDNA and deduced amino acid sequence of PtCHS. A red square box highlights the initial codon and the stop codon.

The cloned PtHMGR gene was 2323 bp long, including the 5′ and 3′ untranslated regions (UTRs). The open reading frame (ORF) was flanked by a 157 bp 5′-UTR and a 384 bp 3′-UTR. PtHMGR had a 1782 bp ORF that encoded 593 amino acids (Fig 12). PtCHS gene had 1633 bp. A 228 bp 5′-UTR and a 235 bp 3′-UTR flanked the ORF. PtCHS had a 1170 bp ORF that encoded 389 amino acids (Fig 13). The molecular formula of PtHMGR was C_2781_H_4486_N_770_O_853_S_33_ with a calculated molecular weight of 63.4 kDa and a theoretical pI of 6.09. The instability index is often used to predict protein stability. If it is less than 40, the protein is considered stable; otherwise, it is considered unstable [41]. The instability index (II) of PtHMGR is 35.54 (<40). Therefore, this protein were therefore classified as stable. Alanine was the most abundant amino acid in protein, accounting for 9.9% of the total amino acids. The PtHMGR and PtCHS sequences are S1 and S2 Files.

**Fig 14:**
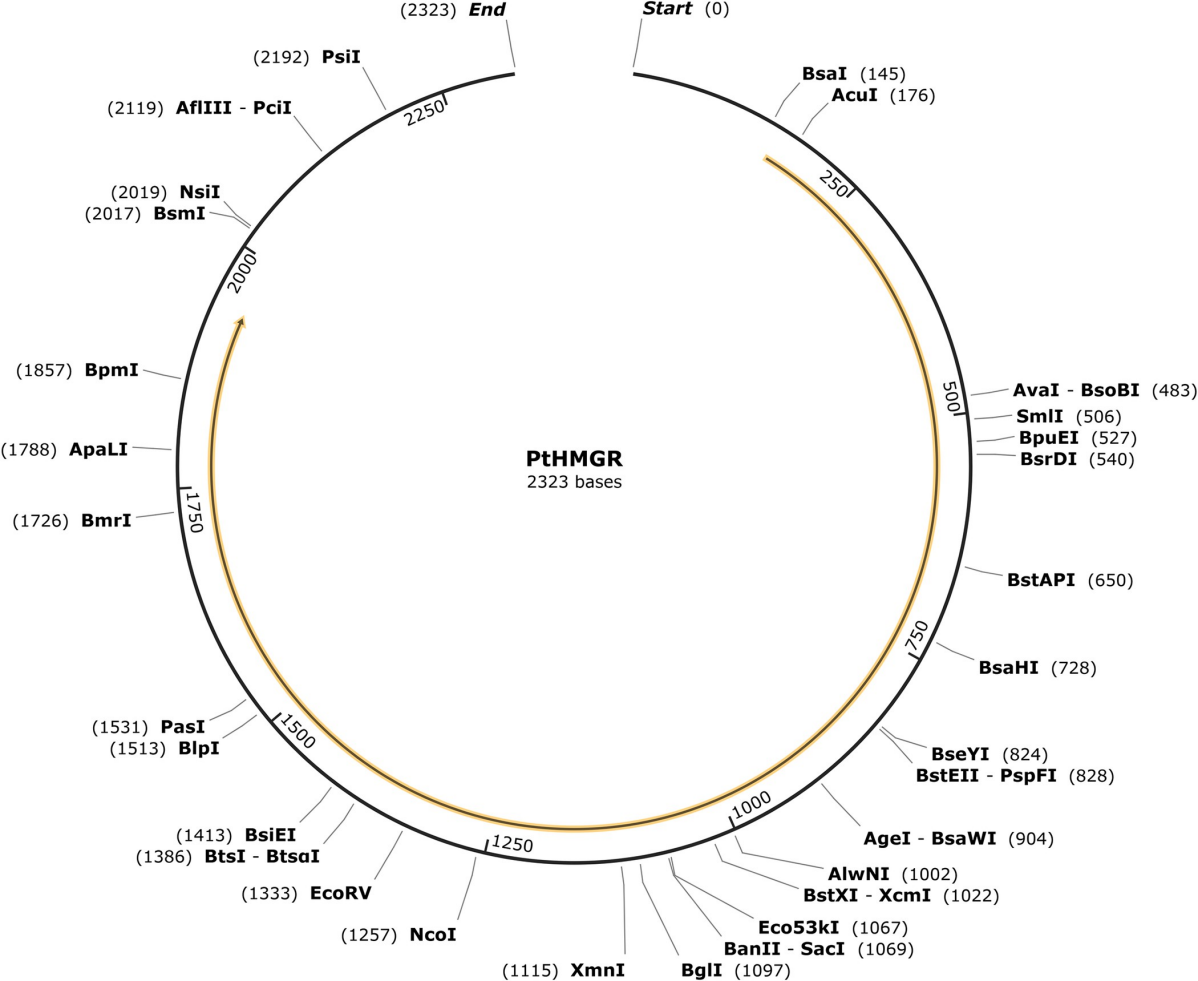
Full length PtHMGR gene and cleaved sites.

**Fig 15:**
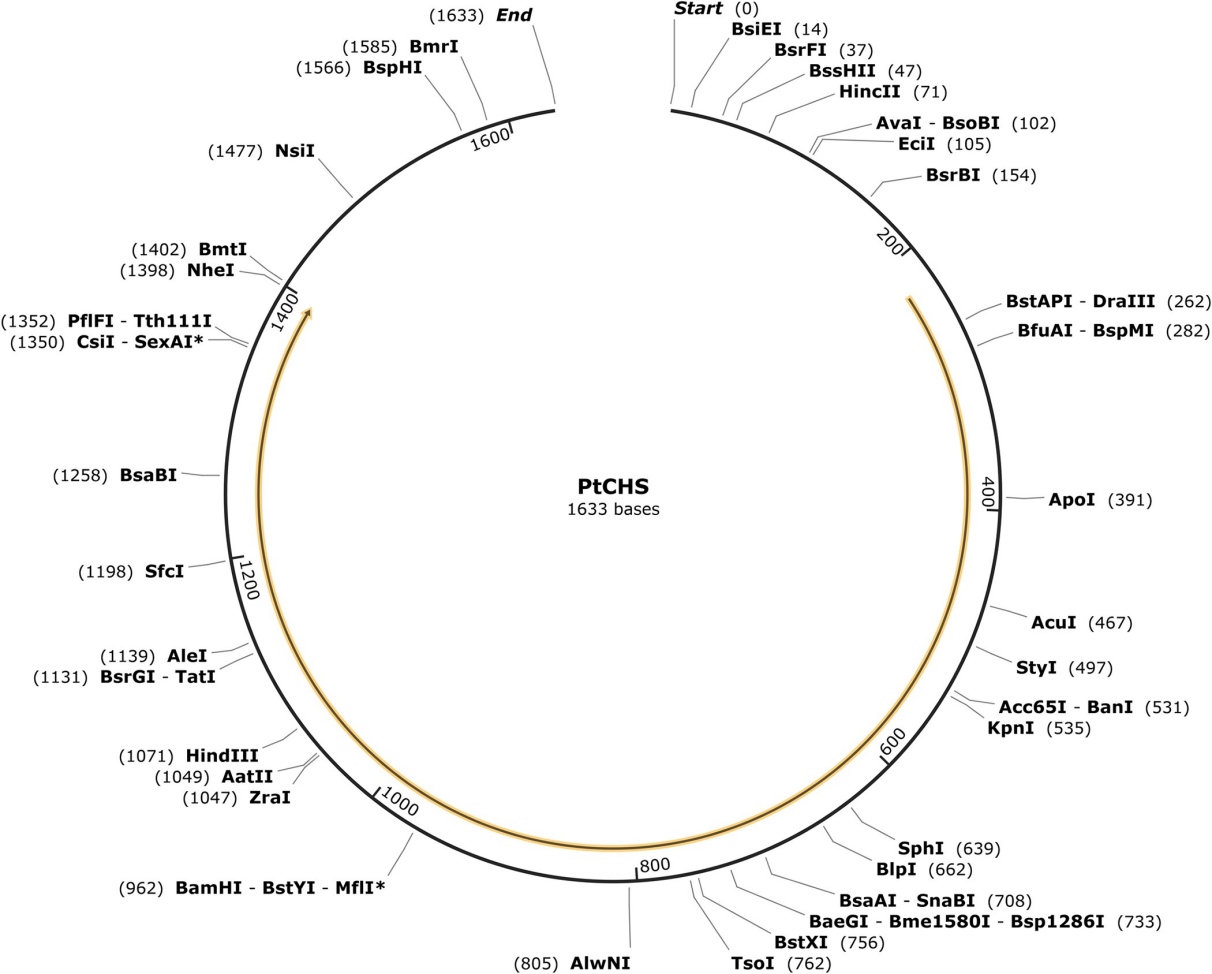
Full length PtCHS gene and cleaved sites.

The molecular formula of *PtCHS* is C_1891_H_3012_N_498_O_560_S_14_, with a molecular weight of 42.1 kDa and a theoretical pI of 5.86. The instability index (II) of PtCHS was 35.29 (<40), therefore, it was classified as stable. Alanine was the most abundant amino acid, encoded 9.0% of the total amino acids in the protein. The PtHMGR gene had 30 cleavage sites (Fig 14), including BsaⅠ, AcuⅠ, SmlⅠ, BglⅠ, PsiⅠ, etc., which were distributed along the entire length of the gene. The PtCHS gene had 36 cleavage sites (Fig 15), including BsiF Ⅰ, BsrF Ⅰ, Apo Ⅰ, Hind Ⅲ, Bmr Ⅰ, etc., which were also distributed throughout the gene length. The enzyme sites of the both genes were rich and could be cleaved by different enzymes in molecular experiments.

### Conserved motifs of PtHMGR and PtCHS

**Fig 16:**
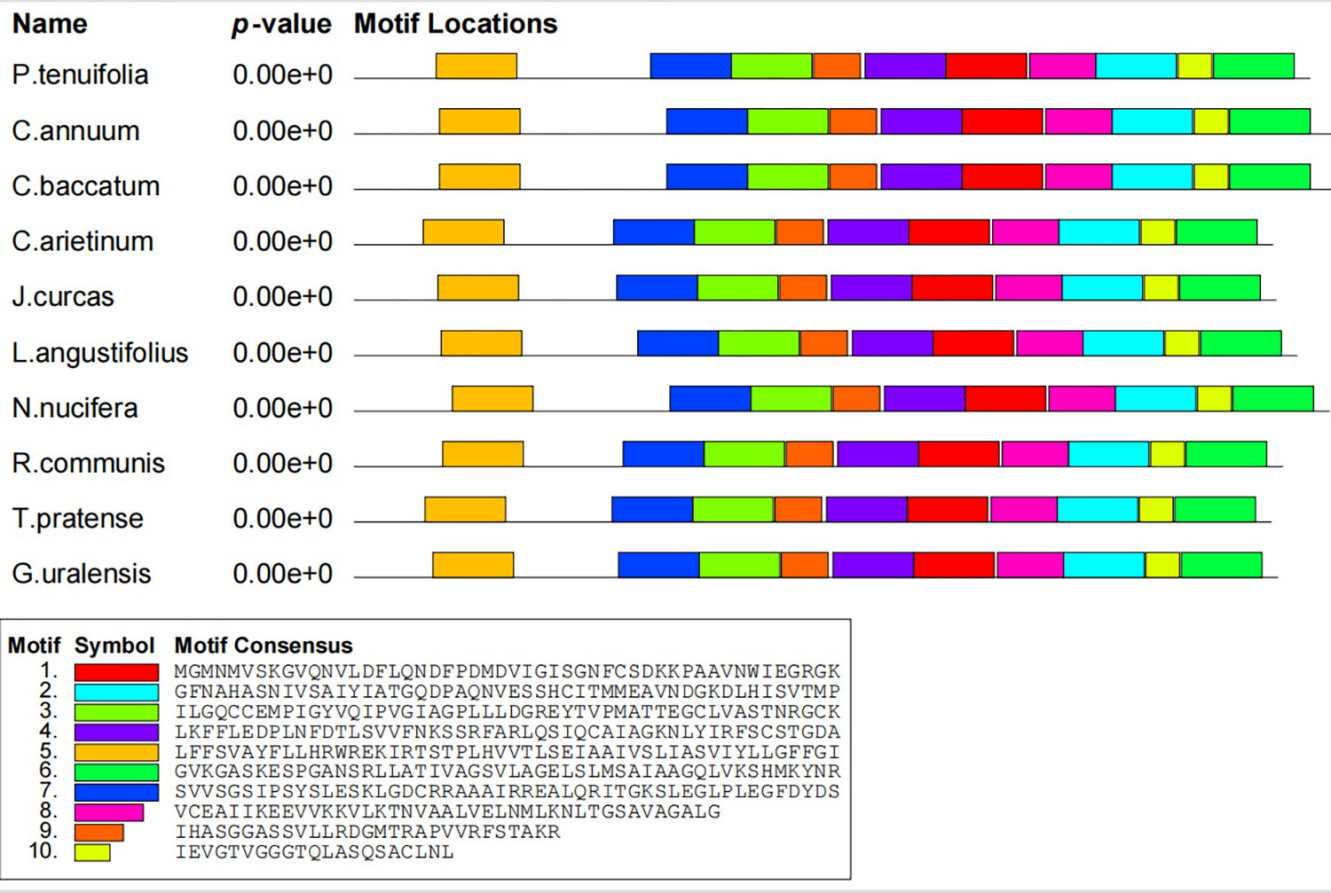
Distribution of conserved motifs of HMGR proteins.

**Fig 17:**
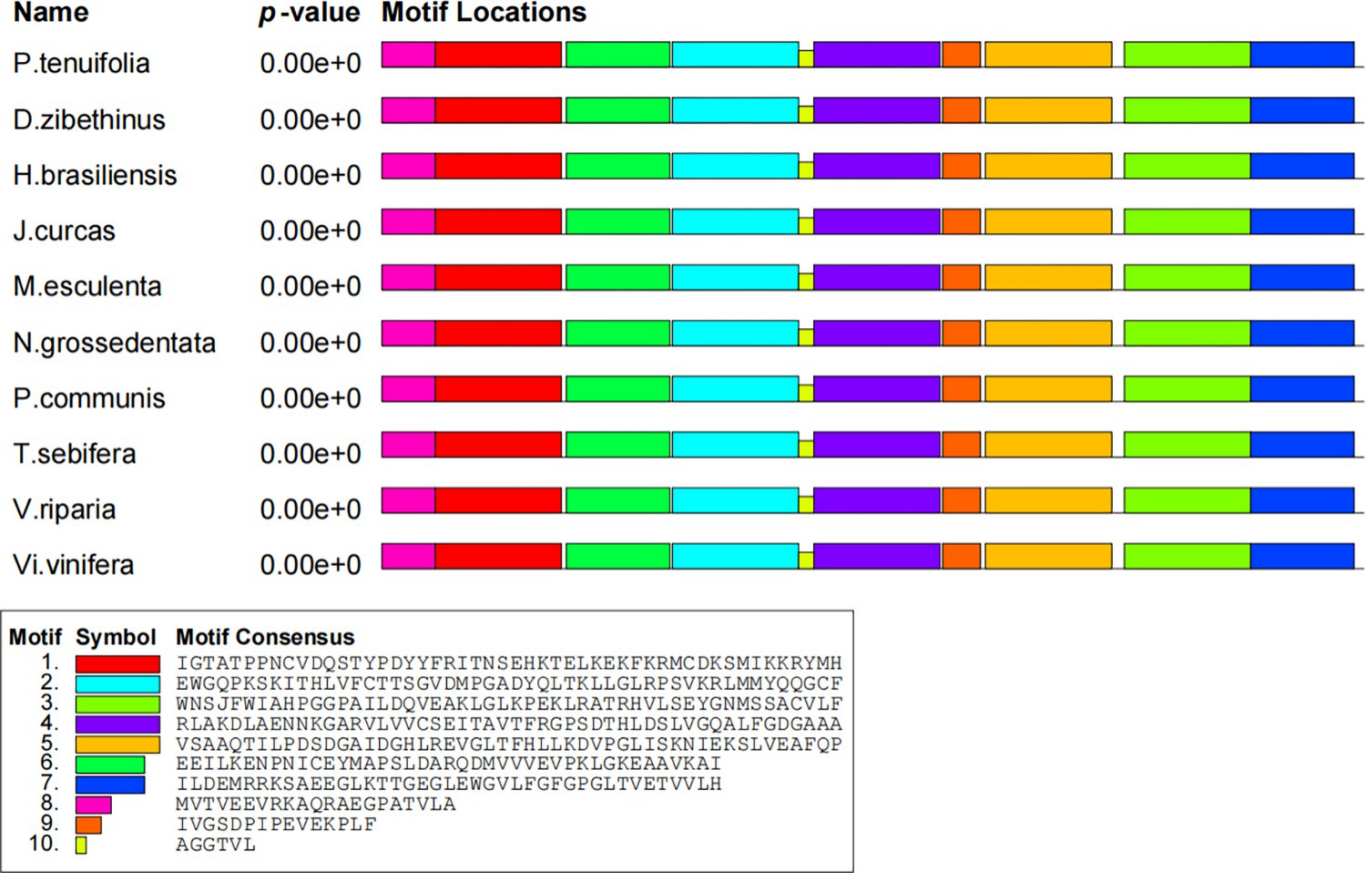
Distribution of conserved motifs of CHS proteins.

To confirm the domains of PtHMGR and other plant HMGR genes, the NCBI Conserved Domains online tool was used. The HMG-CoA reductase class I domain was found in all the HMGR genes. PtHMGR was identified as a 3-Hydroxy-3-methylglutaryl coenzyme A reductase gene. PtCHS also shares the conserved domain with other plant CHS genes and was proved to be a chalcone synthase gene. Simultaneously, the MEME software was used to examine the conserved motifs of the HMGR and CHS proteins. The results showed that all ten HMGR or CHS proteins contained motifs 1~ 10 (Figs 16 and 17). The motif matches showed a position p-value less than 0.0001.

### Phylogeny of PtHMGR and PtCHS

The amino acid sequences of PtHMGR and PtCHS were aligned with those of other plants, the results showed the sequence similarity of PtHMGR was 75–79% with HMGR from other plant species using Blastx/Blastp algorithm, including *Glycyrrhiza uralensis* (79%; Accession No.: ADM87300.1), *Jatropha curcas* (78%; Accession No.: XP_012087580.1), *Trifolium pratense* (78%; Accession No.: PNX95266.1) and *Nelumbo nucifera* (75%; Accession No.: XP_010270571.1). The amino acid sequence of *PtCHS* shares 77–78% identity with CHS from other plant species such as *Vitis rotundifolia* (78%; Accession No. ACN30003.1), *Coffea arabica* (78%; Accession No. XP_027118978.1), *Manihot esculenta* (77%; Accession No. XP_021608720.1), and *Hevea brasiliensis* (77%; Accession No. XP_021648729.1). Multiple amino acid sequences of HMGR and CHS were analyzed between *P*. *tenuifolia* and other plants with DNAMAN V9.0, there were highly conserved sequences between both of them.

**Fig 18:**
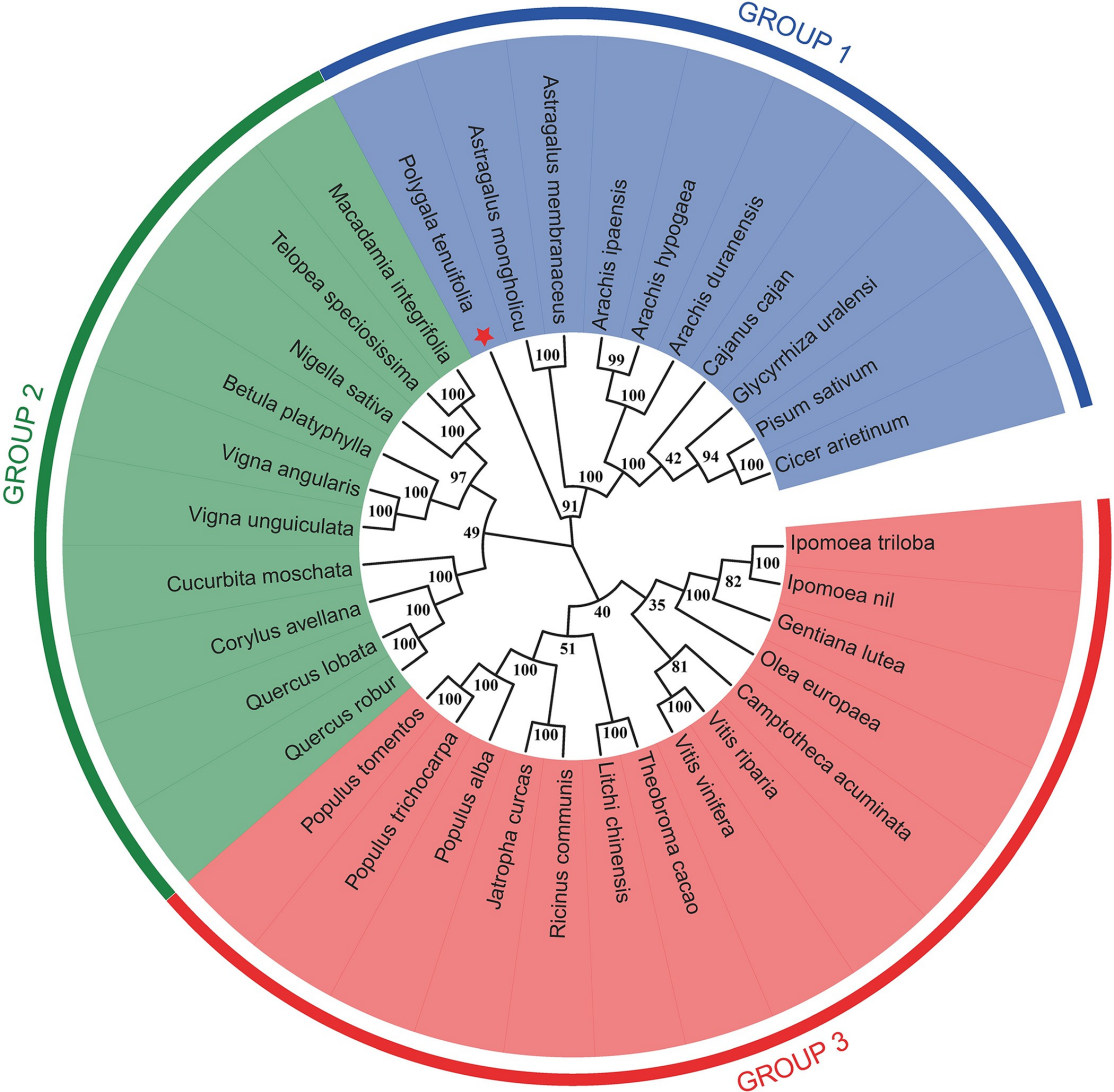
Phylogenetic tree of the PtHMGR gene.

**Fig 19:**
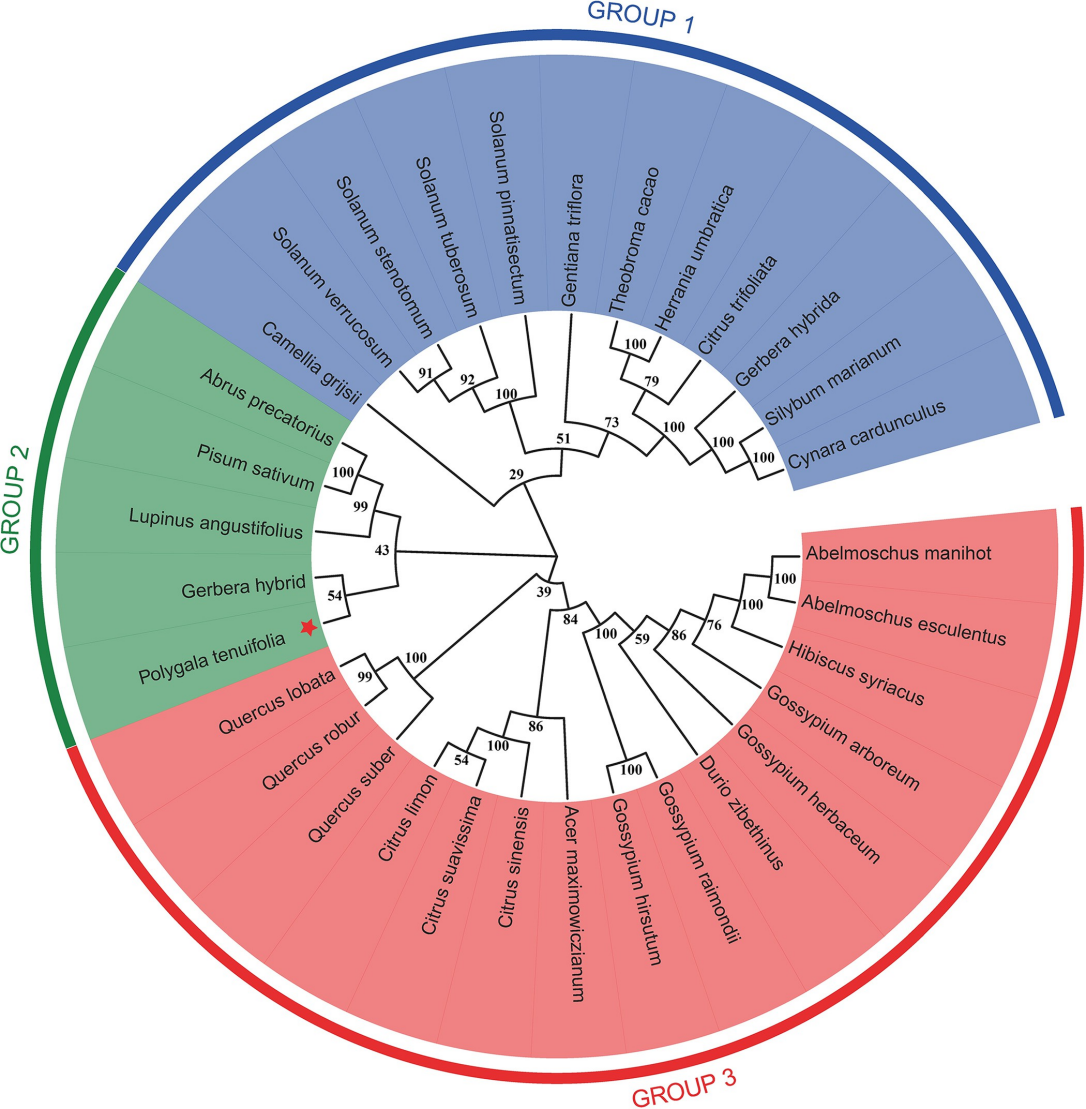
Phylogenetic tree of the PtCHS gene.

Based on the multiple amino acid sequences, phylogenetic trees of PtHMGR (Fig 18) and PtCHS (Fig 19) were constructed by MEGA 11 software using the ML method. *PtHMGR* was included in the first branch with 34 genes. Three phylogenetic groups (groups 1–3) were identified based on phylogenetic analysis. Of the 14 genes, Group 3 had the highest number of members. There were separately 10 genes in Group 1 and group 2. *PtHMGR* was in group 1, clustered with *Astragalus mongholicu*, *Astragalus membranaceus*, *Arachis ipaensis*, *Arachis duranensis*, *Cajanus cajan*, *Glycyrrhiza uralensi*, *Pisum sativum*, *Cicer arietinum*, but the genetic distance was relatively larger than other HMGRs. In 32 CHS genes, three phylogenetic groups were identified (groups 1–3). Group 3 had the highest number of members, including 15 genes. Group 2 had the least number of members, with only 5 genes, and group 1 contained 12 genes. PtCHS was in Group 2, on an evolutionary line with *Gerbera hybrids*, and clustered with *Lupinus angustiffolius*, *Pisum sativum*, *and Abrus precatorius*. The genetic distance was also relatively larger than that of other CHSes. Therefore, PtHMGR or PtCHS is homologous to HMGR or CHS, however, its phylogenetic relationship is far from that of other plants.

### Protein properties of PtHMGR and PtCHS

The hydrophilicity and hydrophobicity of PtHMGR and PtCHS were analyzed with ProtScale. The results showed that the hydrophobicity of the 17th amino acid of PtHMGR was the strongest, which was -3.700. The 86th amino acid of PtHMGR showed the strongest hydrophilicity (2.900). PtCHS showed the strongest hydrophobicity in the 51st amino acid (-2.478), and the strongest hydrophilicity in the 342st amino acid (2.789). The hydrophobicity and hydrophilicity of proteins are determined based on grand average of hydropathy (GRAVY) value, which is positive for hydrophobic protein, and negative for hydrophilic proteins [42]. The GRAVY value of PtHMGR was 0.096. This meant that the hydrophobic region was more than the hydrophilic region, indicating that PtHMGR is a hydrophobic protein. PtCHS is a hydrophobic protein, with a GRAVY value of 0.007. The signal peptides of PtHMGR and PtCHS were predicted with SignalP 4.1 Server, and their scores of the signal peptide and the comprehensive shear site were approximately 0.1, which proved that there was no signal peptide or its cleavage sites. PtHMGR and PtCHS are not secreted proteins. The same prediction results of HMGR were obtained for *Hevea brasiliensis*, *Capsicum annuum*, *Andrographis paniculata* and *Tripterygium wilfordii*. PtHMGR and PtCHS may be synthesized in the cytoplasm, retained in the cytoplasmic matrix without protein transport, and directly interact with metabolic substrates.

Two transmembrane domains in the N-terminus of PtHMGR were predicted with TMHMM Server v2.0, located at 39 ~ 61 aa and 82 ~ 104 aa, respectively. However, PtHMGR is not a signal peptide, and does not undergo the transmembrane operation. PtHMGR is "anchored" to specific parts of the cytoplasmic matrix and exerts a catalytic function between its transmembrane domain and membrane lipid [43]. PtCHS has no transmembrane region, is synthesized in the cytoplasm, and interacts directly with metabolic substrates in the cytoplasmic matrix to exercise their catalytic function [44].

### Secondary and tertiary structure of PtHMGR and PtCHS

Secondary structures of PtHMGR and PtCHS were predicted with SOPMA. The results showed that both of them mainly included α-helices and random curls. PtHMGR contained 274 α-helices, accounting for 46.21%, 88 extended strands (14.84%), 36 β-turns (6.07%), and 195 random-coils (32.88%). The solvent accessibility of PtHMGR was analyzed by PredictProtein, the results showed the exposed protein residues were at 35.41%, intermediate at 8.60%, and buried at 55.99%. PtCHS was composed of 169 α-helices (43.44%), 66 extended strands (16.97%), 23 β-turns (5.91%), and 131 random-coils (33.68%) in secondary structure. The exposed protein residues of PtCHS accounted for 30.85%, intermediate 11.57%, and buried 57.58%. Proteins with higher hydrophobicity are more emulsible and less soluble [45]. PtHMGR and PtCHS are hydrophobic proteins with more buried protein residues and fewer exposed residues, which may influence their functional activities, such as catalysis or binding [46].

**Fig 20:**
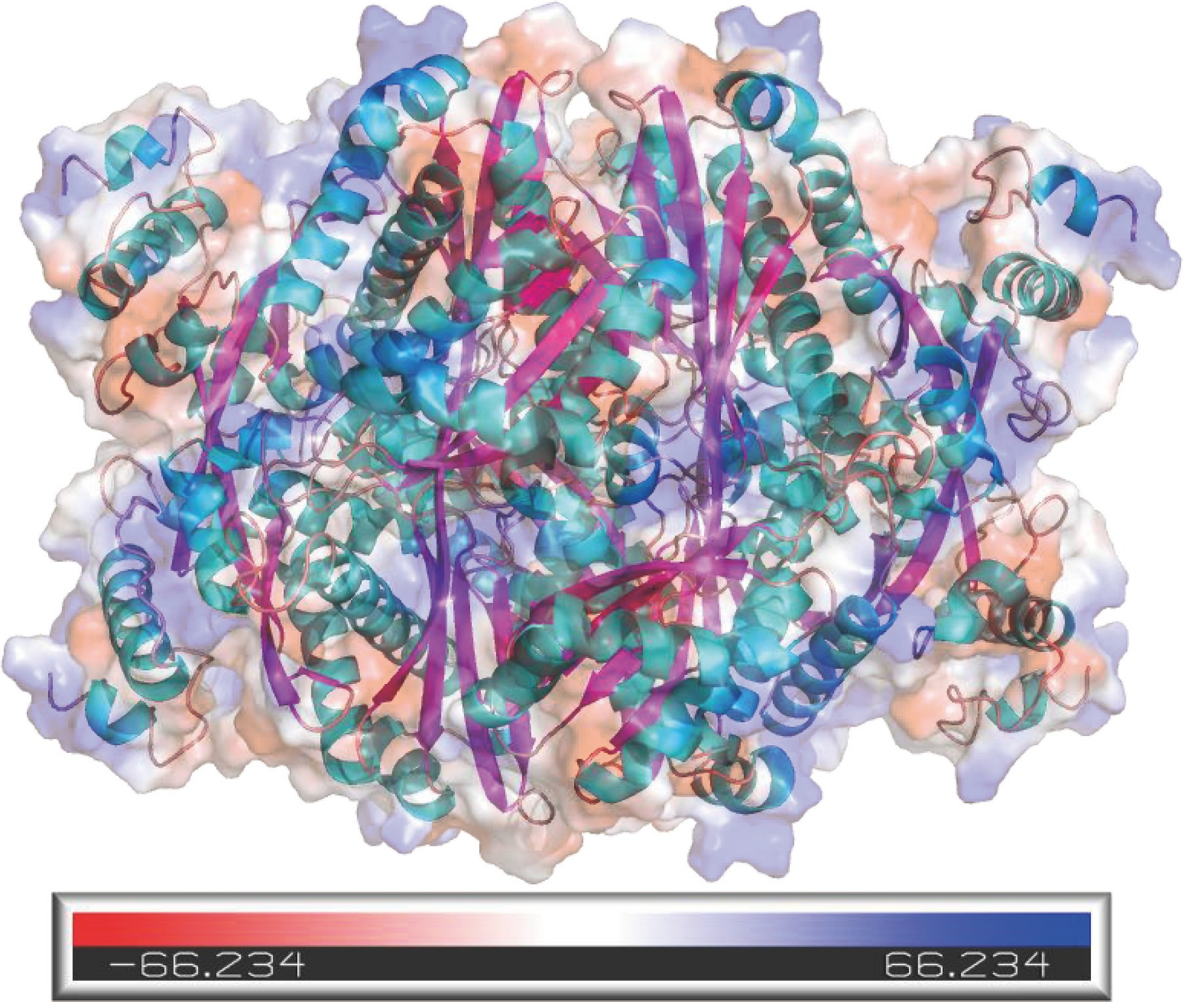
The surface potential distribution of PtHMGR tertiary structure.

**Fig 21:**
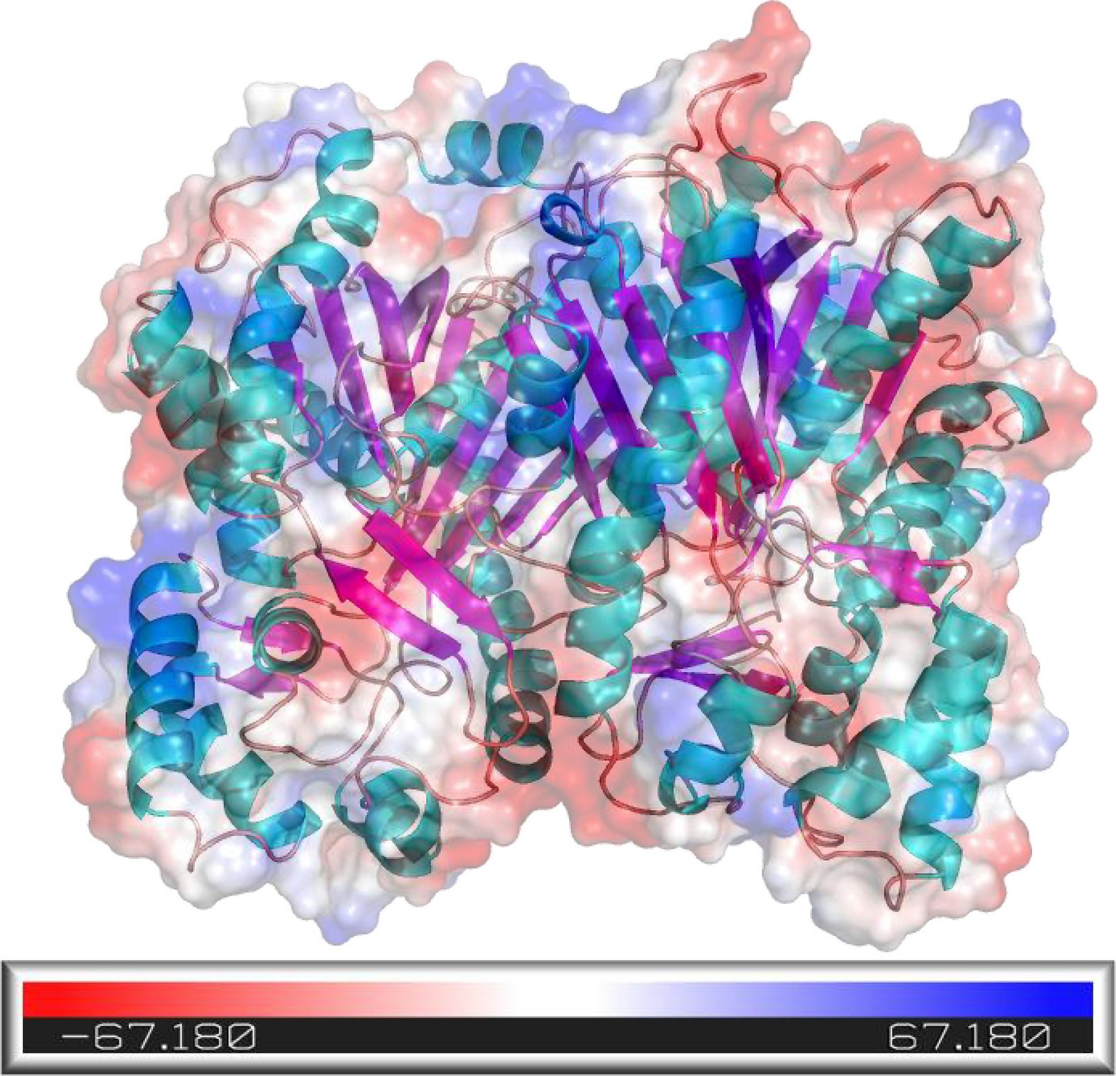
The surface potential distribution of PtCHS tertiary structure.

The tertiary structure and surface potential distribution of PtHMGR (Fig 20) and PtCHS (Fig 21) were obtained by SWISS-MODEL and PyMOL. The colored regions can be used to evaluate the electrophilic and nucleophilic potential of the tertiary structures, and white, blue, and red separately represented neutral, nucleophilic, and electrophilic centers,respectively [47,48]. The PtHMGR color codes ranged from -66.234 a.u. (red) to 66.234 a.u. (blue). And PtCHS color codes were between -67.180 a.u. (red) and 67.180 a.u. (blue). These positive and negative regions may help bind to the reaction substrate [49]. Molecular docking is mediated by electrostatic interactions between the cationic and anionic membrane surfaces [50]. The charge and potential distribution in the catalytic/binding region of the protein may be influenced by the protonation state of residues close to the active site. The charge/potential of the active site area of enzymes determines the degree of interaction between the reaction substrates and/or products if they carry charge [51]. Using NADPH as a hydride donor, HMGR catalyzes the four-electron reduction of thioester (S)-HMG-CoA to primary alcohol (R)-mevalonate in most organisms [52]. According to the InterProScan prediction results, the substrate-binding domain of HMG-CoA on PtHMGR is located at 173–572 amino acid residues. The binding domain of NAD/NADP on PtHMGR is 300–416 residues. Similarly, CHS is a plant-specific type III polyketide synthase that converts one p-coumaroyl-CoA and three malonyl-CoA molecules into 2’, 4, 4’, 6’-tetrahydroxychalcone (THC) [53]. The structural domains of PtCHS were located at 5–228 (N-terminus) and 238–387 (C-terminus) amino acid residues.

**Fig 22:**
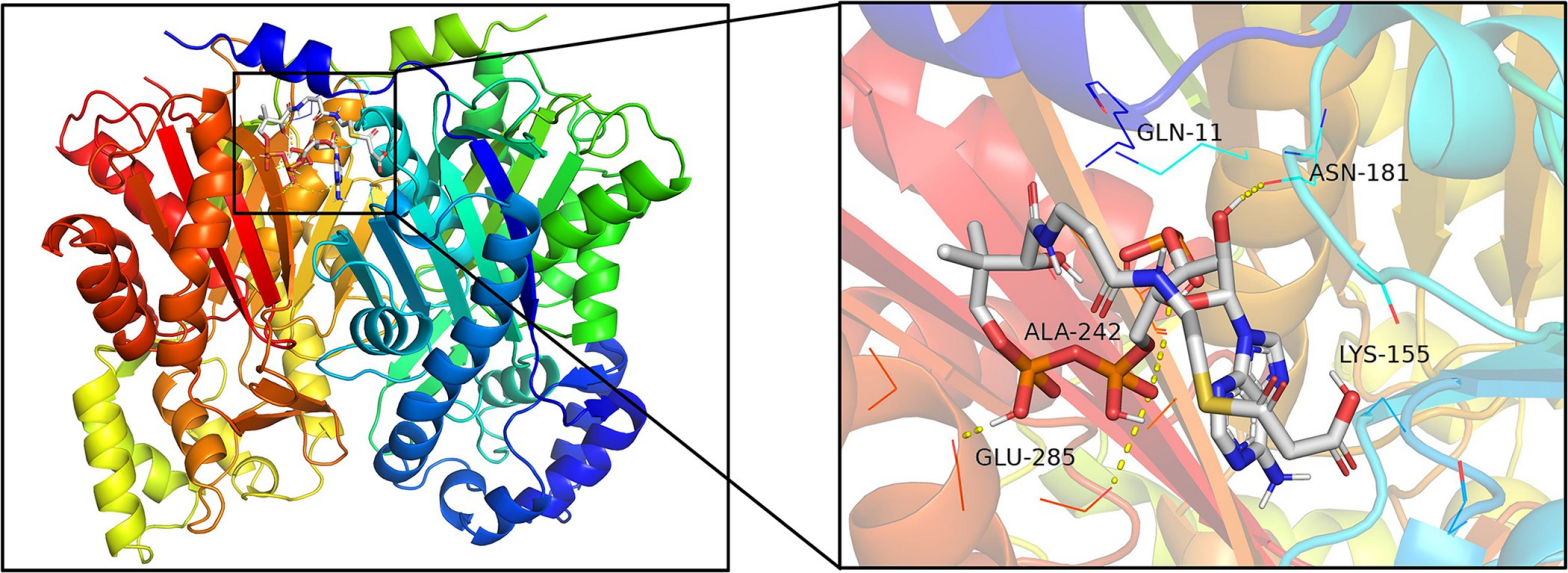
Molecular docking results of PtHMGR and HMG-CoA.

**Fig 23:**
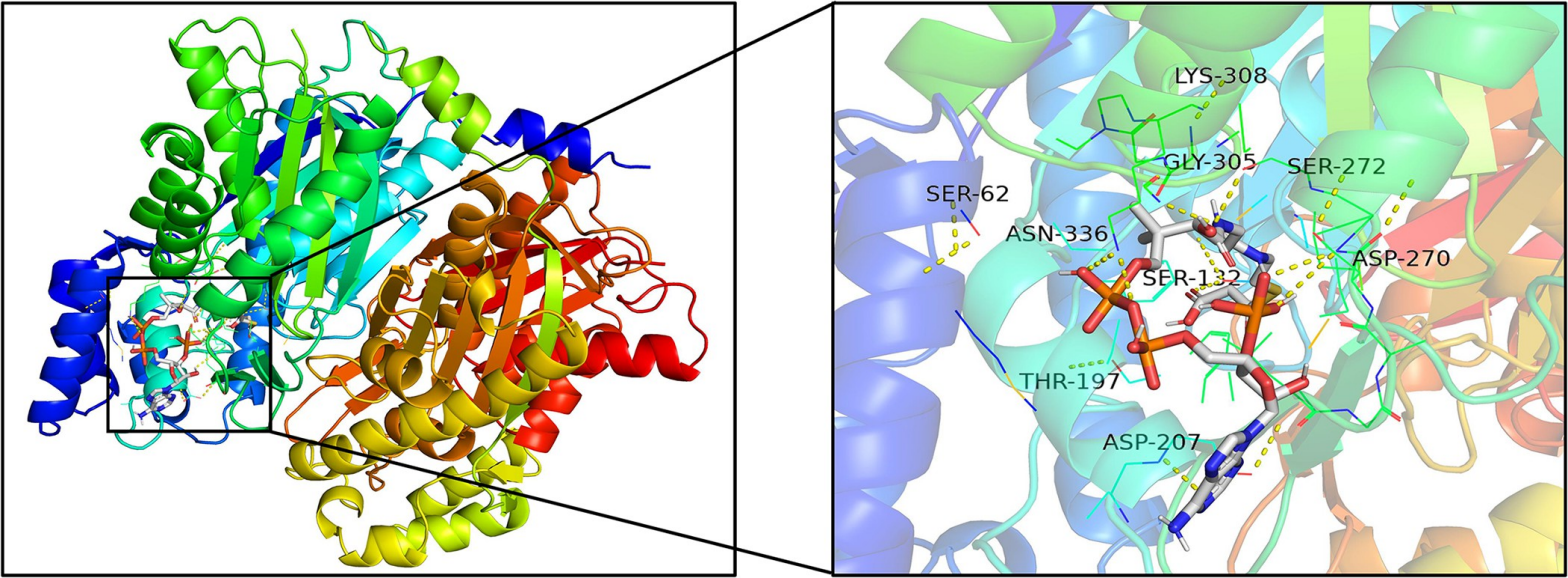
Molecular docking results of PtCHS with malonyl-CoA.

**Fig 24:**
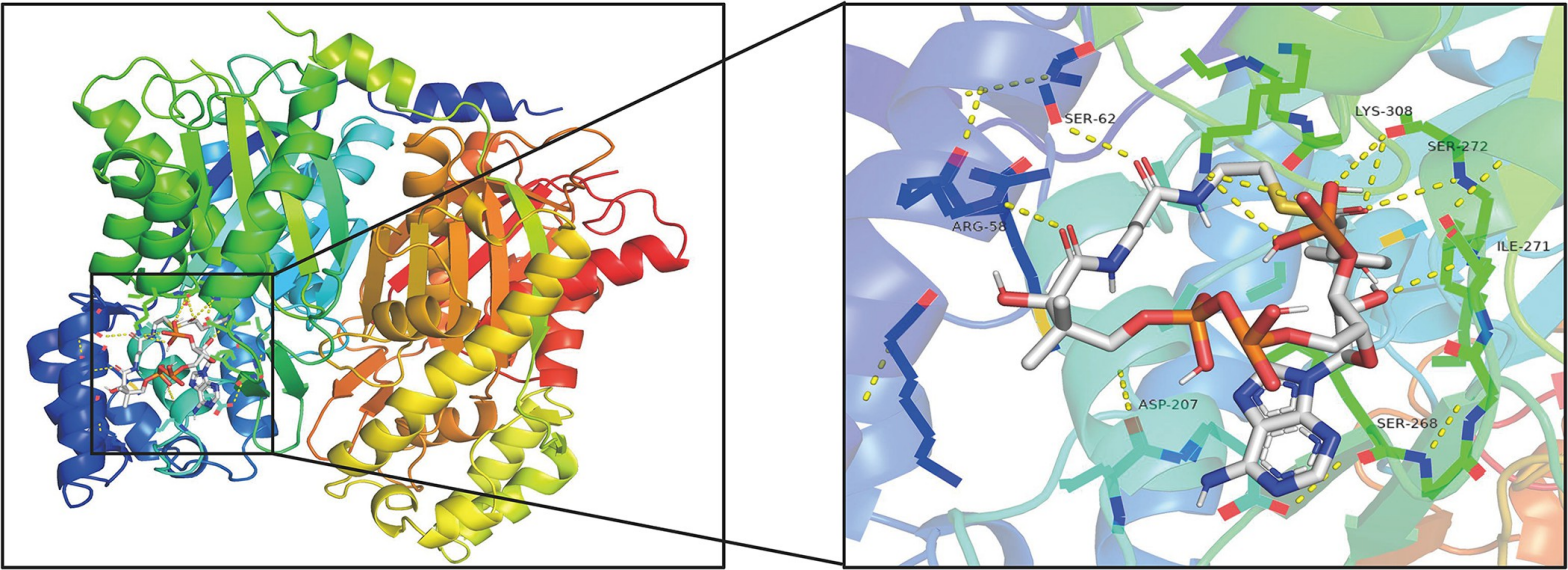
Molecular docking results of PtCHS with 4-coumaroyl-CoA.

Using *Arabidopsis thaliana* HMGR (ID: 7ULI in RCSB PDB) and *Medicago sativa* CHS (ID: 1I86 in RCSB PDB) as templates, the tertiary structures of PtHMGR and PtCHS were modeled by SWISS-MODEL software. The results (Figs 22–24) showed that the main structures of PtHMGR and PtCHS were α-helices, β-turns, and random-coils, and the directions from the N-terminus to the C-terminus are shown in red, orange, yellow, green, cyan, blue, and purple, respectively. 390 amino acids of PtHMGR were highly similarity with to template 7ULI, which had a coverage rate of 92.0%. PtHMGR contained 21 α-helices and 13 β-strands in its tertiary structure and had a complicated spatial architecture that was comparable to that of human HMGR [54]. In plants, HMGR has two motifs (TTEGCLVA and EMPVGYVQIP) that bind to HMG-CoA and two motifs (DAMGMNM and GTVGGGT) that are linked to NADP(H) [55]. The analysis results of CB-Dock2 showed that the binding energy of protein-ligand complex was -6.8 for PtHMGR. PtHMGR residues that bind to substrate HMG-CoA are displayed in Fig 22 with PyMOL software, which were GLN11, LYS155, ASN181, ALA242, and GLU285, three of which were consistent with the report in reference [54].

388 amino acids in PtCHS were highly similar with to template 1I86, with a coverage rate of 100%. PtCHS has 15 α-helices and 18 β-strands in its tertiary structure. The spatial architecture of PtCHS was similar to that of *Rhododendron×hybridum* Hort [56] and *Freesia hybrida* [57]. The CHS-family signature sequence, G(F/L)GPG, was also included in the PtCHS [58]. The binding energies of PtCHS with malonyl-CoA and 4-coumaroyl-CoA were separately -6.5 and -7.4. Fig 23 shows nine binding sites of PtCHS and malonyl-CoA, including SER62, SER132, THR197, ASP207, ASP270, SER272, GLY305, LYS308, and ASN336. Fig 24 displayed seven binding sites of PtCHS and 4-coumaroyl-CoA, which are composed of ARG58, SER62, ASP207, SER268, ILE271, SER272, and LYS308.

### Tissue-specific expression of PtHMGR and PtCHS genes

**Fig 25:**
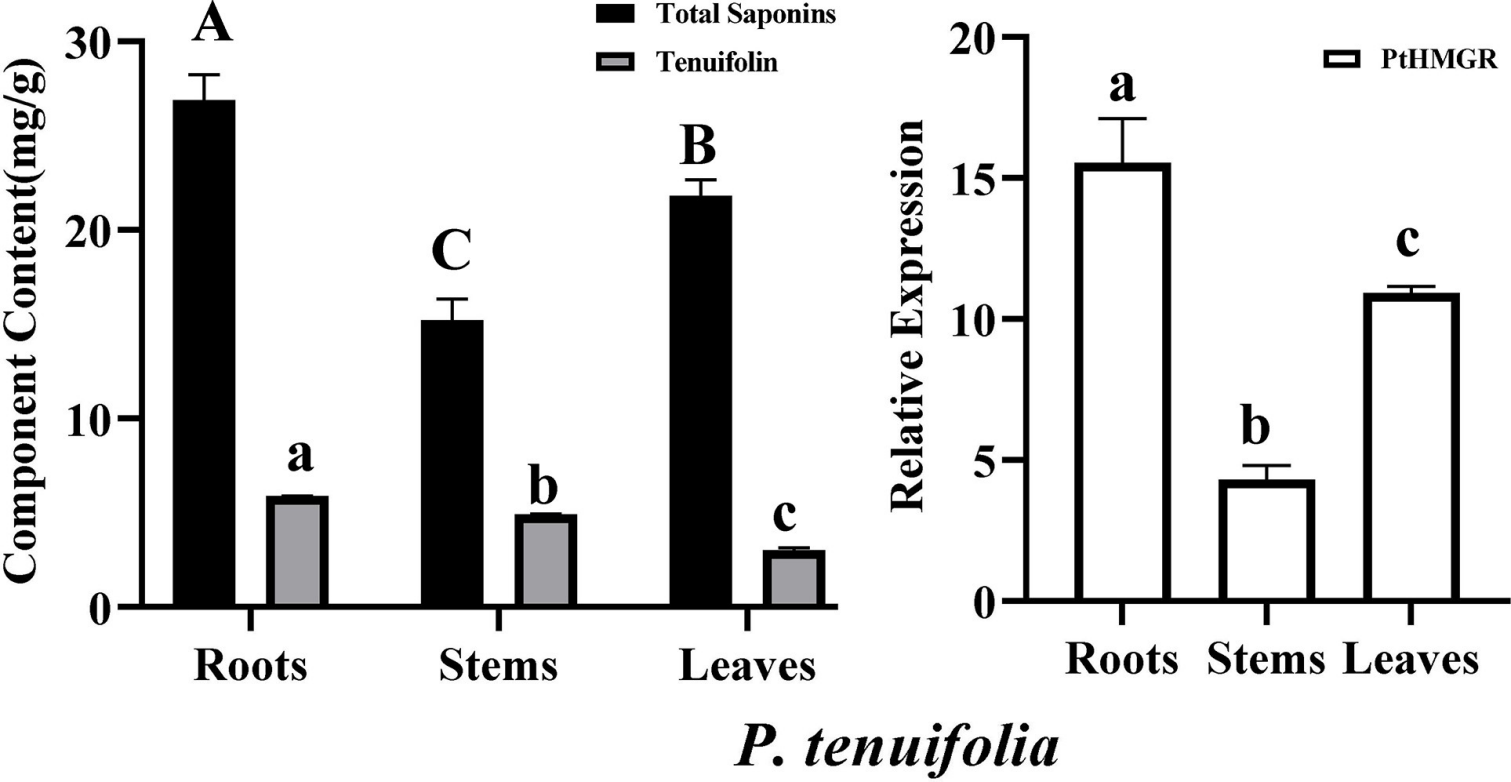
PtHMGR gene expression, total saponin content in root, stem and leaf of *P*. *tenuifolia*. Bars represented mean value ± standard deviation (n = 3). Different letters indicated significantly difference from different tissues (*p* < 0.01).

**Fig 26:**
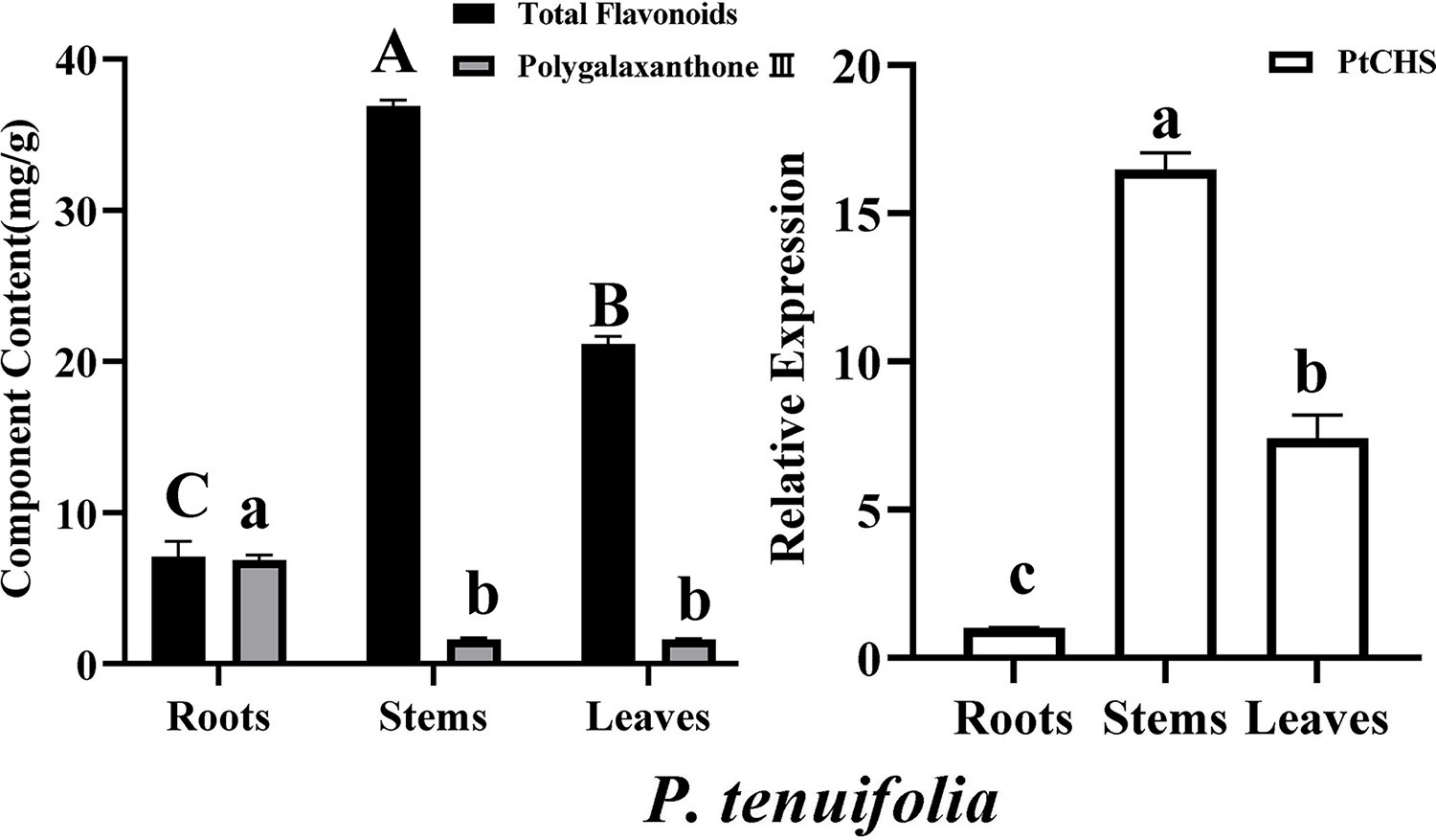
PtCHS gene expression, total flavonoid content in root, stem and leaf of *P*. *tenuifolia*. Bars represented mean value ± standard deviation (n = 3). Different letters indicated significantly difference from different tissues (*p* < 0.01).

The expression levels of the PtHMGR and PtCHS genes were detected by qRT-PCR. As shown in Figs 25 and 26, both PtHMGR and PtCHS genes were expressed in the roots, stems and leaves (Table 3). Their expression varied in different tissues and had significant tissue specificity (p<0.01), which was also consistent with the reported results [59–62]. Stems showed the highest PtCHS expression, about 16.47-fold higher than in root, and about 2.22-fold higher than in leaves (Fig 26). The highest expression of PtHMGR gene was found in the root, followed by the leaf, and the loeest was detected in the stem, where the gene expression level of the root was 3.61- and 1.42-fold higher than those of the stem and leaf, respectively (Fig 25).

**Table 3:** The expression of PtHMGR and PtCHS genes in roots, stems and leaves of *P*. *tenuifolia*.

Part	PtHMGR			PtCHS		
	Content	Mean value	SD	Content	Mean value	SD
Roots	14.58	15.54	1.57	1	1	0.02
	14.68			1.02		
	17.35			0.98		
Stems	4.75	4.31	0.51	15.82	16.47	0.57
	4.42			16.88		
	3.75			16.71		
Leaves	10.66	10.92	0.24	6.82	7.41	0.79
	10.98			7.12		
	11.12			8.3		

### Saponin and flavonoid contents and their correlations with PtHMGR and PtCHS

The standard curves of rutin and senegenin were established, and their linear regression equations were as follows: Y = 8.3265X-0.0318 (R^2^ = 0.9991) with a the linear range of 35–70 μg/mL, used for the determination of total saponin content; Y = 3.4743X + 0.007 (R^2^ = 0.9992) with a linear range of 25–150 μg/mL, used for the determination of total flavonoid content. The total saponin and flavonoid content varied in roots, stems, and leaves of *P*. *tenuifolia* (Figs 25 and 26 and Table 4). The total saponin content was 26.89±1.34 mg/100g in root, 15.22±1.12 mg/100 g in stem and 21.82±0.83 mg/100 g in leaf. The total flavonoid content was 7.09±1.02 mg/100 g in root, 36.90±0.38 mg/100g in stem and 21.15±0.50 mg/100g in leaf. Therefore, saponins mainly accumulated in the roots, and the flavonoids were found in the stems of *P*. *tenuifolia*.

**Table 4:** Content of total saponin and total flavonoid in roots, stems and leaves of *P*.*tenuifolia*.

Part	Total saponins			Total flavonoids		
	Content	Mean value	SD	Content	Mean value	SD
Roots	27.68	26.89	1.34	7.88	7.09	1.02
	25.34			5.94		
	27.65			7.45		
Stems	15.56	15.22	1.12	37.01	36.90	0.38
	16.13			36.48		
	13.97			37.21		
Leaves	21.88	21.82	0.83	21.69	21.15	0.50
	20.96			20.70		
	22.62			21.06		

The standard curves of tenuifolin and polygalaxanthone III were established, their linear regression equations were as follows: Y = 7918.5X + 5.7073 (R^2^ = 0.9993) with the linear range of 0.025–0.4 μg/mL, used for the determination of tenuifolin content; Y = 19459X - 345.5 (R^2^ = 0.9996) with the linear range of 0.029–0.464 μg/mL, used for the determination of polygalaxanthone III content. The content of tenuifolin or polygalaxanthone III varied in the roots, stems and leaves of *P*. *tenuifolia* (Figs 25 and 26 and Table 5). The contents of tenuifolin were separately 5.88±0.02 mg/100 g in root, 4.90±0.09 mg/100 g in stem and 3.02±0.10 mg/100 g in leaf. The content of polygalaxanthone III were separately 6.86±0.32 mg/100g in root, 1.61±0.04 mg/100g in stem, and 1.59±0.06 mg/100g in leaf. Hence, tenuifolin and polygalaxanthone III were highly enriched in the roots and accumulated less in the leaves and stems of *P*. *tenuifolia*.

**Table 5:** Contents of Tenuifolin and polygalaxanthone III in roots, stems and leaves of *P*.*tenuifolia*.

Part	Tenuifolin			Polygalaxanthone III		
	Content	Mean value	SD	Content	Mean value	SD
Roots	5.89	5.88	0.02	6.50	6.86	0.32
	5.88			7.12		
	5.86			6.96		
Stems	4.91	4.91	0.09	1.72	1.61	0.04
	4.94			1.56		
	4.87			1.55		
Leaves	2.99	3.02	0.10	1.59	1.59	0.06
	2.94			1.65		
	3.14			1.53		

Correlation analysis was conducted between the PtHMGR and PtCHS genes and their associated metabolites. The results showed that the expression level of PtHMGR was significantly positively correlated with the total saponin and tenuifolin contents, with the correlation coefficients of 1.000 and 0.998,respectively. The expression level of PtHMGR was synergistic with the total saponin and tenuifolin content. Considering that HMGR is one of the rate-limiting enzymes involved in the saponin biosynthesis in plants, the increase of PtHMGR gene might be an effective approach to promote the higher production of triterpenoid saponins in *P*. *tenuifolia*, which was also confirmed in *Gossypium hirsutum* [63], *Glycyrrhiza uralensis* [64], *Paris fargesii* [65] and *Panax notoginseng* [66].

The gene expression of PtCHS was significantly positively correlated with the total flavonoid content (r = 0.998), which is beneficial for the production of flavonoids. Similar findings have been reported in *Eucommia ulmoides* [60], *Brunfelsia acuminata* [67], *Pyrus communis* [68] and *Citrus sinensis* [69]. However, the polygalaxanthone III content was significantly negatively correlated with PtCHS gene expression (r = -0.811). Polygalaxanthone III belongs to the xanthones, a type of flavonoids that might have its own special biosynthetic pathway [70], and is different from the general flavonoid biosynthesis.

## Conclusion

In the present study, the PtHMGR and PtCHS genes were successfully cloned and characterized from *P*. *tenuifolia*. Sequence alignment and phylogenetic analysis showed that the PtHMGR and PtCHS proteins were highly similar to other known HMGR and CHS proteins. The HMG-CoA-binding sites had five amino acid residues in the tertiary structure of PtHMGR, containing GLN11, LYS155, ASN181, ALA242, and GLU285. The tertiary structure of PtCHS had nine sites that combined with malonyl-CoA, including SER62, SER132, THR197, ASP207, ASP270, SER272, GLY305, LYS308, and ASN336, and seven binding residues with 4-coumaroyl-CoA, including ARG58, SER62, ASP207, SER268, ILE271, SER272, and LYS308. The tissue-specific expression showed that the expression of the PtHMGR gene was highest in the roots of the plant and lowest in the stems. PtHMGR transcripts were significantly positively correlated with total saponin and tenuifolin content. PtCHS transcripts were highly expressed in stems, least in roots, and significantly positively correlated with total flavonoids content, which provided support for total flavonoid production in plants. However, PtCHS showed a significant negative association with polygalaxanthone III content. Polygalaxanthone III is a type of flavonoids that may have its own unique biosynthetic pathway that is different from other flavonoid compounds. The expression of PtHMGR and PtCHS genes increased the content of corresponding metabolites, indicating that PtHMGR and PtCHS play important roles in the response to saponin and flavonoid accumulation. This study provides a theoretical basis and molecular resource for improving the medicinal properties of *P*. *tenuifolia*.

## Supporting information

S1 Raw images Original gel image. (PDF)

S1 File PtHMGR sequence. (FASTA)

S2 File PtHMGR sequence. (FASTA)
