# Supplementary material for: HMGR and CHS gene cloning, characterizations and tissue-specific expressions in Polygala tenuifolia Willd
Source: PLoS One. 2024 Mar 25;19(3):e0300895. doi: 10.1371/journal.pone.0300895 (PMC10962832; doi:10.1371/journal.pone.0300895)

Fig 2 A Total RNA electropherogram of *P. tenuifolia* root

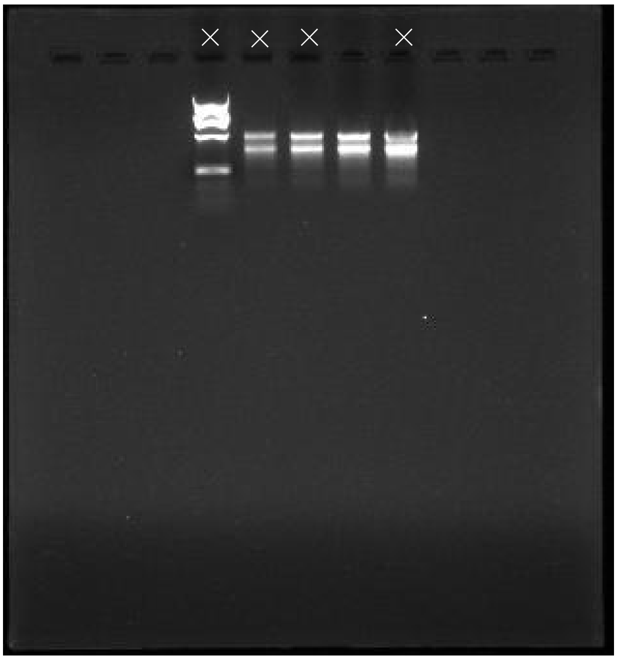

Fig 2 B (a) The electropherogram of intermediate fragments of PtHMGR gene

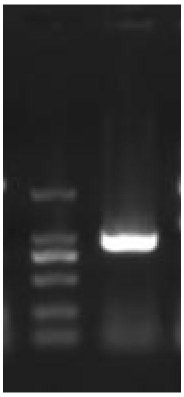

Fig 2 B (b) The electropherogram of intermediate fragments of PtCHS gene

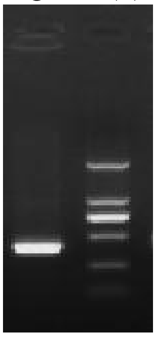

Fig 2 C (a) 5'RACE electrophoretogram of PtHMGR gene

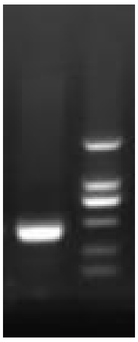

Fig 2 C (b) 5'RACE electrophoretogram of PtCHS gene

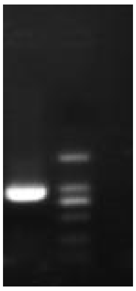

Fig 2 D (a) 3'RACE electrophoretogram of PtHMGR gene

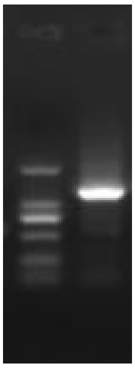

Fig 2 D (b)3'RACE electrophoretogram of PtCHS gene

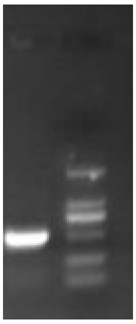

Fig 2 E (a)The electropherogram of full length fragments of PtHMGR gene

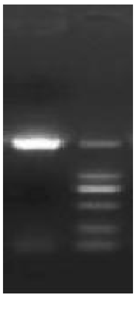

Fig 2 E (b)The electropherogram of full length fragments of PtCHS gene

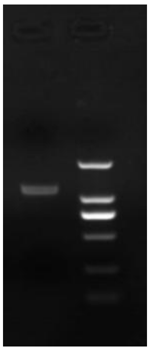

Supplement: S1 Raw images — (PDF) [file pone.0300895.s001.pdf]
